# A Source Apportionment and Emission Scenario Assessment of PM_2.5_‐ and O_3_‐Related Health Impacts in G20 Countries

**DOI:** 10.1029/2022GH000713

**Published:** 2023-01-04

**Authors:** M. Omar Nawaz, Daven K. Henze, Susan C. Anenberg, Caleb Braun, Joshua Miller, Erik Pronk

**Affiliations:** ^1^ Department of Mechanical Engineering University of Colorado Boulder Boulder CO USA; ^2^ Milken Institute School of Public Health George Washington University Washington DC USA; ^3^ Climate Cabinet San Francisco CA USA; ^4^ The International Council on Clean Transportation San Francisco CA USA

**Keywords:** adjoint, modeling, PM_2.5_, O_3_, source, G20

## Abstract

Exposure to air pollution is a leading risk factor for premature death globally; however, the complexity of its formation and the diversity of its sources can make it difficult to address. The Group of Twenty (G20) countries are a collection of the world's largest and most influential economies and are uniquely poised to take action to reduce the global health burden associated with air pollution. We present a framework capable of simultaneously identifying regional and sectoral sources of the health impacts associated with two air pollutants, fine particulate matter (PM_2.5_) and ozone (O_3_) in G20 countries; this framework is also used to assess the health impacts associated with emission reductions. This approach combines GEOS‐Chem adjoint sensitivities, satellite‐derived data, and a new framework designed to better characterize the non‐linear relationship between O_3_ exposures and nitrogen oxides emissions. From this approach, we estimate that a 50% reduction of land transportation emissions by 2040 would result in 251 thousand premature deaths avoided in G20 countries. These premature deaths would be attributable equally to reductions in PM_2.5_ and O_3_ exposure which make up 51% and 49% of the potential benefits, respectively. In our second application, we estimate that the energy generation related co‐benefits associated with G20 countries staying on pace with their net‐zero carbon dioxide targets would be 290 thousand premature deaths avoided in 2040; action by India (47%) would result in the most benefits of any country and a majority of these avoided deaths would be attributable to reductions in PM_2.5_ exposure (68%).

## Key Points

- Fine particulate matter is not always the pollutant most associated with trans‐boundary health impacts; this depends on country and sector
- Reducing land transportation emissions by 50% would result in 251 thousand premature deaths avoided in Group of Twenty (G20) countries in 2040
- Around 300 thousand premature deaths could be avoided in G20 countries if energy emissions reduced on pace with net‐zero carbon dioxide target years

## 1 Introduction

Globally, an estimated 6.7 million (Murray et al., 2020) premature deaths each year are attributable to fine particulate matter (PM_2.5_) and ozone (O_3_) exposure of which 4.5 million are attributable to ambient exposure; most of these premature deaths occur in regions that are in exceedance of the current World Health Organization air quality standards (World Health Organization, 2021). These standards have recently been revised to be more stringent. To address this air‐pollution related health burden, policy‐makers develop regionally‐ (European Union Parliament, 2008; Nakićenović & Swart, 2000; UNEP, 2015), nationally‐ (DEFRA, 2022; National Clean Air Programme, 2019; State Council, 2018; US EPA OAR & US EPA OP, 1999), and locally‐ (Breathe London, 2020; C40 Cities, 2019; Garcetti, 2019) focused policies; however, in doing so they must grapple with a question that is difficult to address from both a scientific and implementation standpoint: “what sources contribute the most to the formation of air pollution?”

The Group of Twenty (G20) countries are a collection of the world's largest economies that formed in response to the 2008 financial crisis. According to the European Commission (European Commission, 2022), the G20 member states contributed around 90% of the global gross domestic product and made up over 60% of the global population (G20 Foundation, 2017) in 2020. These dominant economies play an integral role in a majority of air‐pollution‐related premature deaths; ambient pollution exposure in G20 countries results in over 3.0 million premature deaths each year (Murray et al., 2020) as estimated in the Global Burden of Disease (GBD) 2019 study while consumption based emissions from G20 countries were responsible for 1.9 million air‐pollution related premature deaths globally in 2010 (Nansai et al., 2021). In order for G20 countries to develop effective policies to alleviate the health burden of poor air quality, accurate characterizations of the sources of their air pollution are required, along with estimates of the health benefits that would be associated with proposed emission reduction scenarios. Many G20 countries have made national commitments to mitigate climate change (United Nations, 2022) by setting net‐zero carbon dioxide (CO_2_) target years. Mitigation plans aim to replace energy generation from fossil fuel combustion with cleaner energy sources. These transitions to cleaner energy will reduce emissions of pollutants and pollutant precursors (P. Wang et al., 2021) resulting in co‐benefits of improved air quality and public health.

A challenge in identifying the sources of air pollution is presented by the inherent complexity of its formation. The chemical precursors of PM_2.5_ and O_3_, along with these pollutants themselves, can originate far upwind of a country and be transported across continents and oceans affecting individuals in entirely different jurisdictions from where they were emitted (Dentener et al., 2010; Dong et al., 2018; Hakim et al., 2019; Holloway et al., 2003; Liang et al., 2018). The formation of air pollution is a product of non‐linear chemical reactions (Aksoyoglu et al., 2017; Campbell et al., 2021; Cohan et al., 2005), dynamics of the atmosphere (Moreira & Vilhena, 2009), and photochemistry (Altshuller & Bufalini, 1971); all of these factors also have seasonal and diurnal dependencies (Song et al., 2018; Wespes et al., 2017). There are many different chemical precursor species that form PM_2.5_ and O_3_. Anthropogenic emissions of primary carbonaceous aerosols like black (BC) and organic (OC) carbon are a direct source of PM_2.5_ (Contini et al., 2018), but PM_2.5_ can also form secondarily in the atmosphere. Secondary aerosols are classified as either inorganic (Fu et al., 2016), which form from emitted ammonia (NH_3_), nitrogen oxides (NO_x_), and sulfur dioxide (SO_2_), or organic (Kroll & Seinfeld, 2008) which form from volatile organic compounds (VOCs) reacting with oxidants in the atmosphere. The anthropogenic precursors that contribute the most to O_3_ pollution are NO_x_ and VOCs. PM_2.5_, especially its primary components, has a shorter lifetime on the order of days to a week than O_3_ which is generally on the order of a month (Seinfeld & Pandis, 2016); owing to this, O_3_ formation can be more dependent on distant sources than PM_2.5_.

Different emission sectors and regions can be linked to unique chemical precursor species; for example, NH_3_ is emitted in large quantities from agriculture and livestock, generally in more rural areas, although recent work has identified that transportation‐related NH_3_ is likely underrepresented (Cao et al., 2022). Another example, NO_x_, is associated in part with vehicle emissions that are of greater magnitude in more urbanized areas. Formal source attribution studies more specifically estimate the contributions of particular emissions to modeled or observed pollution levels using a variety of approaches such as factor analysis (Xie et al., 2013), model tagging (Goldberg et al., 2016), and model sensitivity analyses including both brute force (McDuffie et al., 2021) and adjoint‐based (Nawaz et al., 2021) calculations. Once relationships between pollutants and precursor emissions have been estimated they can be used to project the impact that changes in emissions would have on air pollution (Lacey et al., 2017).

A number of studies have performed source attributions of air pollution and its associated health impacts for multiple countries considering sectors (McDuffie et al., 2021) and regions (Qiao et al., 2019). Sectoral and regional source attributions are generally conducted separately; however, they have been calculated in tandem (Crippa et al., 2016; Van Dingenen et al., 2018) primarily using reduced complexity models. One effort considered the consumption footprint of G20 countries and their impacts on one another (Nansai et al., 2021). Other studies have considered source attributions for a single receptor; this includes work focused on individual countries (Ren et al., 2021), sub‐national regions (Zhang et al., 2015), or cities (Nawaz et al., 2021) with a general emphasis on either regional or sectoral sources but rarely both (H.‐M. Lee et al., 2017). Many source attributions focus on PM_2.5_ as it bears a larger health burden globally and because it involves less non‐linear chemistry than O_3_; for the latter, the role of transport is important due to its longer lifetime (Zawacki et al., 2018). When considering the health burden associated with long‐range transport it is unclear whether the larger health burden of PM_2.5_ or the longer lifetime of O_3_ will prevail as the dominant factor driving long‐range impacts; this is especially true when considering individual regional or sectoral sources.

In our analysis, we characterize how sectoral and regional sources, individually and in tandem, contribute to PM_2.5_‐ and O_3_‐related premature deaths in the 16 G20 countries that are not members of the European Union (EU) and the additional 27 countries of the EU. We consider precursor emissions of BC, OC, NH_3_, NO_x_, SO_2_, VOCs, and carbon monoxide (CO) from the aviation (AIR), agriculture (AGR), energy (ENE), industry (IND), residential (RES), shipping (SHP), and land transportation (TRN) sectors to identify sectoral source contributions. The sensitivities of air pollution in each G20 country to the emissions of different precursor species are calculated using the adjoint of the chemical transport model GEOS‐Chem. We then combine these pollutant‐emission sensitivities, which are calculated for every grid cell in the modeling domain, with emissions, aggregated to the country level, and perform a health impact assessment to estimate how emissions from every country in the world contribute to the air pollution health burden in each of the G20 countries. A new second‐order adjustment to the source contribution calculation is introduced to better characterize the non‐linear relationships between O_3_ and NO_x_. The key results of this study, the regional and species‐specific sensitivities of PM_2.5_ and O_3_ to emissions, are then used to assess the air‐pollution health benefits that could be achieved from different emission scenarios including a 50% reduction in transportation emissions and the emission reduction co‐benefits associated with G20 countries staying on target to achieve their net‐zero CO_2_ goals.

## 2 Methodology

To characterize the sources of PM_2.5_‐ and O_3_‐related premature deaths and to assess health benefits associated with projected changes in emissions we incorporate a number of unique approaches in conjunction. First, we employ air quality modeling and adjoint calculations to characterize the sensitivity of pollutant exposures to emissions of their chemical precursors. These sensitivities are combined with emissions from distinct sectors and regions to estimate source contributions to PM_2.5_ and O_3_ exposures. To assess the health benefits associated with emission reductions, we combine the same sensitivities with proposed emission scenarios to evaluate the impact that changing emissions would have on PM_2.5_ and O_3_ exposures. We include these exposure impacts in concentration‐response relationships and, using population data and baseline mortality rates, estimate the premature deaths contributed from different sources along with the health impacts avoided from different scenarios. Each of these steps, along with a number of assumptions, introduce uncertainty into our results; an extensive discussion of uncertainty is available in the supplemental along with a qualitative summary in Section 3.5.

### 2.1 Emissions

We use two distinct emission inventories for different parts of our analysis. For the air quality modeling with GEOS‐Chem, we use emissions from HTAP v2.2 for a base year of 2010 (Janssens‐Maenhout et al., 2015) of BC, OC, NH_3_, NO_x_, SO_2_, VOCs, and CO. For source attribution, we use these same emissions; HTAP emissions include seven sectors: AIR, AGR, ENE, IND, RES, SHP, and TRN. We use HTAP v2.2 as opposed to ECLIPSE v5a here as the prior is available at a finer resolution of 0.1° × 0.1° than the latter with a resolution of 0.5° × 0.5°. Using a finer resolution inventory makes regional source attribution more accurate by better attributing emissions alongside country borders. For the emission scenario assessments we use emissions from the current legislation scenario (CLE) of ECLIPSE v5a (IIASA, 2021) for a base year of 2040 which includes the same set of precursor species as HTAP sub‐divided into nine sectors: AGR, ENE, IND, RES, SHP, gasoline based on‐road transportation, diesel based on‐road transportation, other non‐road transportation, and waste. For the purpose of our analysis, we aggregate the three TRN sectors in ECLIPSE v5a into a single TRN sector. The emission scenarios, discussed in greater detail in Section 2.5, project emissions to 2040. We use ECLIPSE v5a emissions for these emission scenario assessments because future scenarios are unavailable for HTAP v2.2.

In this work, we aim to create a source receptor data set that can be used broadly and that is not tied to any specific emission inventory. Using an inventory for the source attribution that differs from what was used for the air quality simulation introduces little uncertainty into our analysis; adjoint cost‐function contributions calculated with ECLIPSE emissions agree well with forward model finite difference tests as discussed in greater detail in the Supporting Information S1.

### 2.2 Air Quality Modeling

The formation of PM_2.5_ and O_3_ is simulated using the GEOS‐Chem chemical transport model (Bey et al., 2001) (the adjoint (Henze et al., 2007) version 35 corresponding to version 10 of the standard model) at a horizontal resolution of 2° × 2.5° with 47 vertical layers; we use GEOS‐5 assimilated meteorology for 2010 from the NASA Global Modeling and Assimilation Office (Gass, 2021). This standard simulation of GEOS‐Chem will be referred to as the “forward” model; in this simulation, model sensitivities are calculated forward in time from emissions to pollutant concentrations. For each of the two pollutants and 43 G20 countries we perform 12 simulations of 2‐months in length; we perform 2‐month simulations, as opposed to single month simulations, to ensure there is a sufficient spin‐up period and to account for emissions impacts from earlier months in the adjoint calculation. For each of the twelve 2‐month simulation we only consider concentrations from the second month to compute our exposure metrics; for PM_2.5_ we consider annual averaged population‐weighted concentrations in 2010 and for O_3_ we consider the population‐weighted 6‐month peak maximum daily 8‐hr average concentrations in 2010. Overall, over 1,000 2‐month simulations were performed to generate the results presented in this work. In this version of GEOS‐Chem we do not consider fugitive dust and secondary‐organic aerosols. By omitting these two sources, simulated PM_2.5_ is biased low and we underrepresent the sectors associated with these sources while overrepresenting sectors that are not associated with these sources in our source apportionment. These sources were excluded from this work because some of the forward model simulations and adjoint calculations in this study were conducted prior to these updates. It has been estimated that anthropogenic secondary‐organic aerosols account for between 15% and 30% (Nault et al., 2020) of PM_2.5_ in urban areas, primarily from vehicle emissions and volatile chemical products. The inclusion of anthropogenic fugitive, combustion, and industrial dust was estimated (Philip et al., 2017) to increase global population‐weighted PM_2.5_ concentration by 2.9 μg m^−3^ with the largest concentration increases in China and India.

### 2.3 Adjoint Modeling

In the adjoint simulation, we perform a reverse integration of the forward model calculating the sensitivity of the scalar cost‐functions, country‐scale PM_2.5_ and O_3_ exposures, to precursor emissions. For PM_2.5_ we calculate sensitivities to BC, OC, NH_3_, NO_x_, and SO_2_ emissions. For O_3_, we calculate sensitivities to NO_x_, VOCs, and CO. The two cost‐functions are calculated for each country using output from the forward simulation; for PM_2.5_ we also include satellite‐derived data through satellite downscaling and rescaling (C. J. Lee et al., 2015). Through satellite‐derived downscaling we characterize fine‐scale peaks and valleys of concentration in our cost‐function and by rescaling we reduce biases in the simulated concentrations compared to satellite‐derived values. We incorporate satellite‐derived data into our cost‐functions because, in part, the GEOS‐Chem resolution is too coarse to resolve some countries. Through the inclusion of satellite‐derived data, we reduce resolution‐based uncertainty in the health impact assessment; however, we note that the adjoint sensitivities are calculated at the coarser resolution of the model which introduces uncertainty as discussed in the Supporting Information S1. Cost‐function definitions for both PM_2.5_ and O_3_ are provided in our previous work (Nawaz et al., 2021) with two differences: the native model resolution of our calculations here is 2°×2.5°, which is coarser than the urban calculations from our prior work, and we consider cost function domains defined across individual countries. The adjoint model calculates the sensitivities of these cost‐functions to emissions:

(1) $$\lambda_{I,k}=\nabla_{E_{I,k}}J=\frac{\partial J}{\partial E_{I,k}}$$

The adjoint calculates sensitivities ($\lambda_{I,k}$) to emissions ($E_{I,k}$) from every model grid cell, I, and every precursor species, k by taking the gradient of the cost‐function with respect to precursor emissions. Encoded in these sensitivity values are the physical equations, chemical relationships, and meteorological inputs that drive the formation of the pollutants in the forward model captured at the emissions magnitude at which the simulation occurred.

The adjoint sensitivity calculations, compared to equivalent zero‐out forward model sensitivity calculations, are preferable from a computational expense standpoint when sensitivities to multiple different sources for a single receptor are of interest, even when considering results aggregated at the country scale. To obtain the same level of information about source sensitivities calculated in this study, that is impacts from unique combinations of all seven sectors and every country in the world, over 17,000 2‐month forward model simulations would be required.

### 2.4 Source Attribution Calculation

The adjoint sensitivities discussed in the prior section, in the context of our analysis, represent the response of a cost‐function to its precursor emissions. A simple method to perform a source attribution is to multiply emissions from a specific source, $E_{I,k,s}$, with its corresponding adjoint sensitivity, $\lambda_{I,k}$, to estimate the amount that source contributed to the cost‐function $dJ_{I,k,s}$:

(2) $$dJ_{I,k,s}\approx \lambda_{I,k}\times E_{I,k,s}$$

In applying this approach, we assume that the sensitivity—the local‐linear approximation of the response of the cost‐function to its precursor emissions—is representative across large emission changes. Specifically, the sensitivity calculated by the adjoint model is assumed to be representative of the relationship between the cost‐function and emissions regardless of the total emissions amount. This assumption is fine for linear relationships, for example, the sensitivity of PM_2.5_ to emissions of primary carbonaceous aerosols; however, this assumption is weaker for non‐linear relationships between pollutants and precursor emissions, for example, the sensitivity of O_3_ to emissions of NO_x_. An improvement in the characterization of non‐linear relationships can be obtained by assuming that there is a quadratic relationship between the cost‐function and its precursor emissions, that is,:

(3) $$dJ_{I,k}\approx c_{1}E_{I,k}^{2}+c_{2}E_{I,k}+c_{3}$$

We use five unique outputs from our simulations, occurring at specific emission values, to solve for these three coefficients. This is a least squares problem where we intend to solve for a 3×1 vector of coefficients, **m**, with a known 5×3 matrix of emissions values **G**, given outputs from our simulations in the form of a 5×1 vector **d**:

(4) Gm=d

We formulate this as a least squares problem, as opposed to a 3×3 system of equations in order to incorporate additional information from more simulations as discussed in greater detail below. Additionally, we expect this fit to be approximate, not exact, due to the fact that the simulation outputs, **d**, correspond to changes throughout our modeling domain and not just in one country at a time. The matrix **G** includes emissions values from four different scenarios described below, and the derivative of Equation 3 to correspond with the adjoint sensitivities:

(5) $$G=\left[\begin{array}{ccc} {{(E}_{I,k}^{T})}^{2} & E_{I,k}^{T} & 1\\ {{(E}_{I,k}^{N})}^{2} & E_{I,k}^{N} & 1\\ {{(E}_{I,k}^{Z})}^{2} & E_{I,k}^{Z} & 1\\ {{(E}_{I,k}^{D})}^{2} & E_{I,k}^{D} & 1\\ 2E_{I,k}^{T} & 1 & 0 \end{array}\right]$$

The vector **d** includes the cost functions from the four distinct scenarios with a fraction term indicating the amount contributed from a unique grid cell and species based on the first‐order estimates using Equation 2, and the adjoint sensitivities as the fifth element.

(6) $$d=\left[\begin{array}{c} \frac{dJ_{I,k}^{T}}{\sum_{I,k}dJ_{I,k}^{T}}J^{T}\\ \frac{dJ_{I,k}^{N}}{\sum_{I,k}dJ_{I,k}^{N}}J^{N}\\ \frac{dJ_{I,k}^{Z}}{\sum_{I,k}dJ_{I,k}^{Z}}J^{Z}\\ \frac{dJ_{I,k}^{D}}{\sum_{I,k}dJ_{I,k}^{D}}J^{D}\\ \lambda_{I,k} \end{array}\right]$$

We consider four forward model simulations that span a range of regimes: one with the base emission amount indicated by a *T* superscript, one with only natural emissions with anthropogenic emissions from all species removed indicated by a *N* superscript, one with anthropogenic NO_x_ emissions reduced to 10% indicated by a *Z* superscript, and one with double anthropogenic NO_x_ emissions indicated by a *D* superscript. We calculate the fraction contributed by each grid cell and species as estimated by the first‐order approximation and multiply this fraction by the actual cost‐function value calculated from these four forward model simulations. For the fifth element we consider the adjoint sensitivities, which characterize the linear response in cost‐function values with respect to emissions or the first derivative. To calculate the coefficients of the second‐order contribution calculation we can include the adjoint sensitivities as part of the least squares problem by equating them to the derivative of Equation 3. Doing this allows us to simultaneously consider forward model outputs and adjoint sensitivities in our calculation of the second‐order coefficients.

We compute the coefficient vector, **m**, for each 2°×2.5° grid cell and for every O_3_ adjoint calculation for the relationship between O_3_ and NO_x_. For each, **m** is computed as:

(7) $$m={\left(G^{T}G\right)}^{-1}G^{T}d$$

To avoid numerical error, we calculate the Moore‐Penrose pseudoinverse of **G**:

(8) $$G^{†}=V_{p}S_{p}^{-1}U_{p}^{T}$$

and calculate the generalized inverse solution as:

(9) $$m=G^{†}d$$

where **V**_p_ and **U**_p_ are the first *p* right and left singular vectors of **G** and **S**_p_ is a diagonal matrix with elements are the first *p* singular values, where *p* is the rank of **G**.

The performance of the contributions calculated using the second‐order approach is compared to the first‐order approach by performing forward model finite difference tests for single grid cells and comparing them to adjoint contribution calculations from both the first‐ and second‐order approaches. Overall, we find that the second‐order approach for the relationship between O_3_ and NO_x_ agrees better with finite difference tests than the first‐order approach by way of a lower normalized mean bias across the tested grid cells as discussed in Section 3.1.

A few other modifications have been made to the contribution calculation that have yet to be discussed. For first‐order contributions, we scale the total contribution of semi‐normalized sensitivities to the cost‐function values as discussed in our previous study (Nawaz et al., 2021). For the emission scenarios (Section 2.5) we scale PM_2.5_ and O_3_ exposures to match the exposure estimates from the GBD 2019 study (Murray et al., 2020). Additionally, for the source apportionment analysis we scale the O_3_ sensitivities, calculated with a cost‐function of maximum daily 1‐hr max O_3_ to the maximum daily 8‐hr max O_3_ to align our exposure estimate with that of the GBD study; this scaling introduces little uncertainty as discussed in Supporting Information S1.

### 2.5 Emission Scenarios

In a similar manner to how a source attribution is performed, adjoint sensitivities can be combined with changes in emissions from emission scenarios to assess the associated response in pollutant exposures. For this approach, we only apply the first‐order contribution equation discussed in the previous section. Using the first order approach here introduces less uncertainty than for the source attribution as the emission changes considered in scenarios are smaller than the absolute amounts considered in source attributions. However, applying the second‐order approach is possible and an ongoing area of future work. More explicitly, we use the equation:

(10) $$dJ_{I,k,s}=\lambda_{I,k}\times {∆E}_{I,k,s}$$

This is identical to Equation 2 with the exception that we now consider ${∆E}_{I,k,s}$, a change in emissions from an emission scenario rather than the absolute total emissions. We employ the ECLIPSEv5a (IIASA, 2021) inventory in this calculation, as it has emission projections for future years; doing so introduces little uncertainty as discussed in greater detail in the Supporting Information S1. We consider projections to the year of 2040 for both scenarios discussed below and compare reduced emissions to the ECLIPSE v5a CLE for 2040.

Using this approach, we consider two different scenarios. First, we consider how a 50% reduction in air pollutant precursor emissions from the transportation sector would result in reductions of pollutant exposures in 2040 relative to the current legislation projection. We combine a 50% reduction in all transportation emissions with our adjoint sensitivities to assess the health benefits of this action. In the second scenario, we estimate the CO_2_ co‐benefits in the form of air pollution attributable deaths avoided, from G20 countries staying on track to meet their net‐zero CO_2_ emission target years. We reduce emissions from the energy sector based on the difference between 2040 and the net‐zero target year divided by the difference between 2020 and the net‐zero target year.

### 2.6 Health Impacts

Once PM_2.5_ and O_3_ exposure contributions are calculated either by performing a source apportionment (Section 2.4) or through an emission scenario application (Section 2.5) the last step of our analysis is to calculate the health impacts associated with that change in exposure. First, we discuss how we calculate health impacts from exposure to PM_2.5_ and O_3_ in a general sense following the methodology of the GBD 2019 study (Murray et al., 2020). Next, we consider how to assess health impacts from specific exposure contributions. Lastly, we discuss how disease rate and population data are projected to future years. For further details about our approach for the first two steps, we refer to our previous study (Nawaz et al., 2021).

First, we discuss how we estimate the relative risks associated with pollutant exposure levels. For PM_2.5_, the GBD study provides look‐up tables that map annual average population‐weighted PM_2.5_ exposure levels to associated relative risks of premature death from ischemic heart disease, stroke, chronic obstructive pulmonary disorder (COPD), acute lower respiratory illness, lung cancer, and type‐2 diabetes. We note that the relationship between relative risk and PM_2.5_ exposure used in the GBD analysis is more uncertain at high exposures due to a lack of cohort studies and thus our results in countries with high PM_2.5_ exposures have a greater degree of uncertainty. Though we calculate health impacts for each of these outcomes we only present results throughout our analysis as the sum of all individual outcomes. For both PM_2.5_, and O_3_ population‐weighted concentrations we use population data from the Gridded Population of the World data set (CIESIN, 2018). The exposure and relative risk values are mapped at discrete values, as opposed to a continuous function, so we linearly interpolate between points in the look‐up table when exposure values do not line up exactly with look‐up table values. For O_3_, relative risks of premature death from COPD associated with exposure are calculated using a log‐linear model (Jerrett et al., 2009):

(11) $$RR=e^{\beta ∆X}$$

where RR is the relative risk, ∆X is the additional O_3_ increment above the baseline value calculated from a forward model simulation of GEOS‐Chem, and β is the concentration‐response factor which captures the relationship between the risk of premature death from COPD and O_3_ exposure. In this study, we use an estimated relative risk of death from COPD of 1.06 per 10 ppb O_3_ increment and assume a theoretical minimum risk exposure level (TMREL) that is a uniform distribution between 29.1 and 35.7 ppb; both of these values come from the GBD 2019 study (Murray et al., 2020).

Once the relative risks are obtained, premature deaths are estimated by the following equation:

(12) $$∆Mortality=y_{0}\left(1-\frac{1}{RR}\right)Pop$$

where ∆Mortality is the additional premature deaths associated with the relative risk level, $y_{0}$ is the baseline mortality rate for a specific health outcome, and Pop refers to the population exposed to the concentration level. Baseline mortality rates are obtained for each country from the GBD results tool (Murray et al., 2020). Country‐level population data is also obtained from the GBD 2019 study (Murray et al., 2020).

We calculate the health impacts associated with a specific source or scenario contribution by applying the health impact assessment methodology outlined above to two distinct exposure values. First, we consider the baseline exposure values; for PM_2.5_ we use satellite‐derived concentrations (van Donkelaar et al., 2016) as a baseline to estimate the exposure inputs used in the look‐up tables, and for O_3_ we use simulated 6‐month peak maximum daily 8‐hr average O_3_ values for input into Equation 11. Then we estimate the health impacts associated from the baseline exposures with the exposure contribution from a source or scenario estimated using Equation 2 removed from the baseline. When assessing health impacts we consider source contributions from unique sectors, species, and countries; when assessing scenario benefits, we consider gridded contributions of unique species and sectors. By taking the difference in health impacts from these two distinct exposure levels we estimate the health impacts associated with a specific source or scenario.

Lastly, for the emission scenarios in which we project health impacts in future years from reductions in pollutant exposures, we use population and mortality rate projections from the GBD Foresight project (IHME, 2018) for 2040. We performed a rescaling of these mortality rates to be consistent with the GBD 2019 to avoid a substantial disconnect between historical and projected baseline rates. To rescale to values consistent with the GBD 2019, we first calculated the annualized percent change in the GBD foresight mortality rates estimates from 5‐year increments, beginning in 2015 until 2040. Then we matched these to the corresponding country‐cause‐age combination in the GBD 2019 data for 2019. We applied the 2015–2020 annualized rate for one year to calculate 2020 projected values from the 2019 data then we applied the rates for each 5‐year increment beyond 2020 to calculate annual projections of baseline mortality rates to 2040. When we estimate health benefits in future years from changes in emissions, the projected changes in population, age‐demographics, and disease rates, that vary by country, influence our results beyond emission changes alone.

## 3 Results

**Figure 1:**
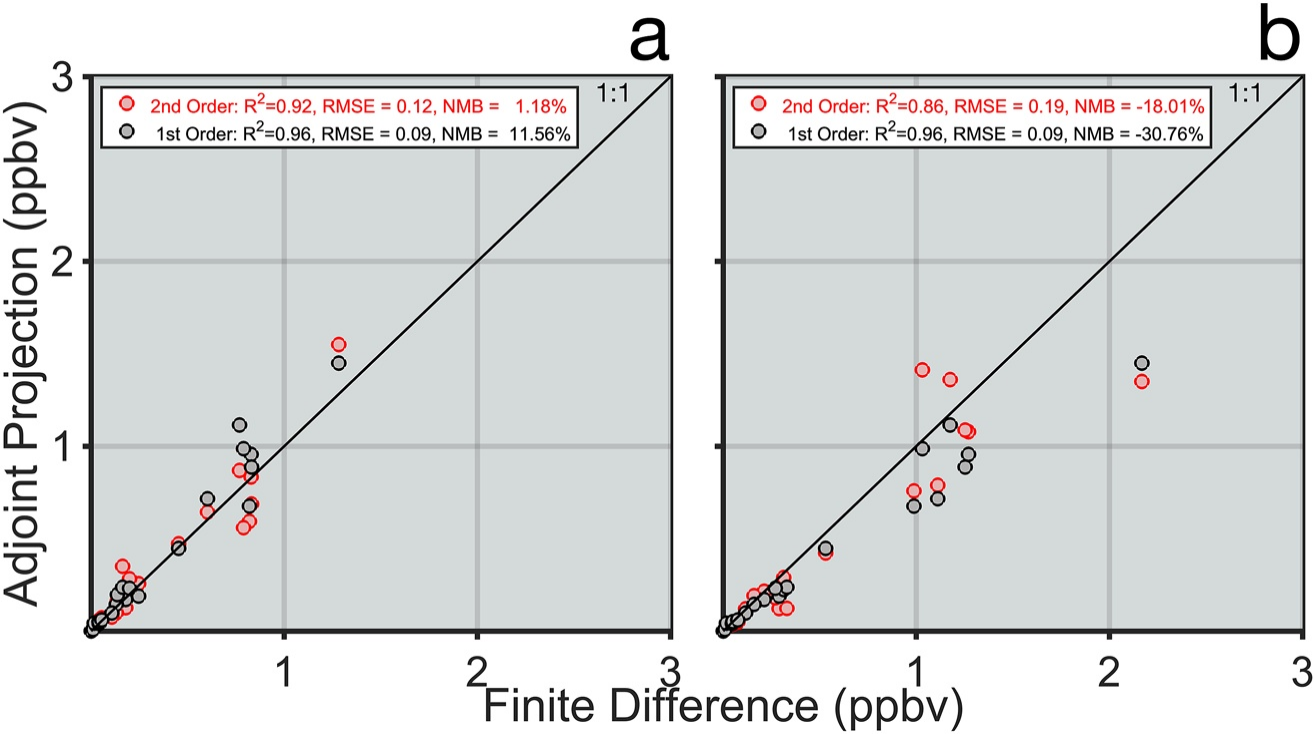
Comparison between the forward model response and first‐order (black) and second‐order (red) adjoint projections of a +50% (a) and −50% (b) change in NO_x_ emissions for the country with the largest response for each of the 21 tests.

**Figure 2:**
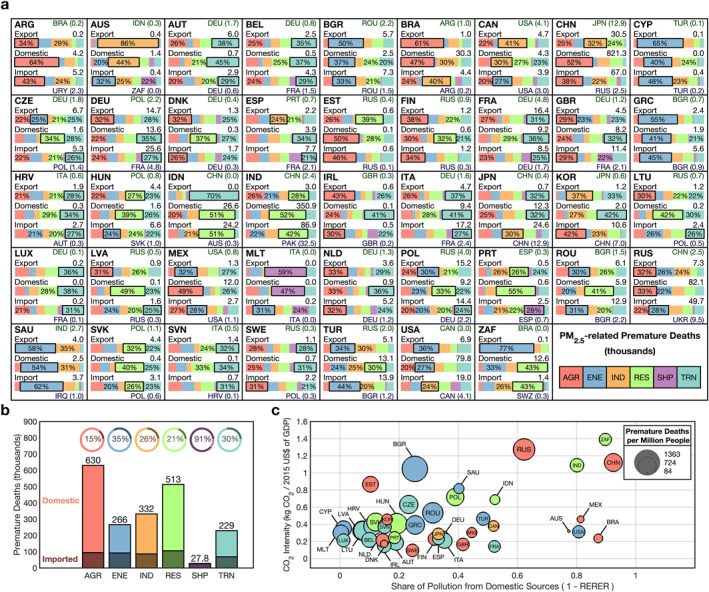
(a) Source contribution summaries of fine particulate matter (PM_2.5_)‐related premature deaths in thousands for every G20 country. A diagram breaking down the figure contents in detail for an example country is in Supporting Information S1. The G20 country is indicated by the ISO code in the top left; a mapping of ISO codes to country names is presented in Table 1. For each country, three bars indicate the exported, domestic, and imported premature deaths; exported deaths refer to deaths in other G20 countries from emissions from the selected country, domestic deaths refer to deaths in the selected country from emissions from the selected country, and imported deaths refer to deaths in the selected country from emissions outside of the selected country. The bars present the normalized breakdown by sector for each of these three source‐receptor relationships with percentages greater than or equal to 20% included on figure. A color map for the sectors is presented in the bottom right of the panel. The absolute number of premature deaths for each of these source‐receptor relationships is given above each of the bars on the right side, across from the abbreviation. The largest source of imported premature deaths from all countries is given in the bottom right in blue. The G20 country most affected by the country's emissions, outside of the country, is given in the top right in green; only G20 countries are considered in exported premature deaths. A country's total premature deaths can be calculated by adding the domestic and imported totals; a country's total health burden impact on all other G20 countries is equivalent to the exported premature deaths. All totals presented are the central estimate, but lower and upper bounds are available in the data set associated with this work. (b) Total G20 PM_2.5_‐related premature death contributions in thousands from all major sectors. All sectors but aviation, which only contributes a small amount to the global health burden (1%) are presented with the absolute amount contributed from each sector above each of the bars. We consider agriculture (AGR), energy (ENE), industry (IND), residential (RES), shipping (SHP), and transportation (TRN). The darker component of each of the bars represents the imported premature deaths while the lighter component represents the domestic premature deaths. The normalized breakdown of imported and domestic contributions is presented in the charts above each of the bars with the percentage indicating the fraction of all contributions from imported sources. (c) Bubble chart comparison of CO_2_ intensity versus the share of pollution from domestic sources for PM_2.5_‐related per‐capita‐premature deaths in all G20 countries. The size of each bubble indicates the premature deaths per million people from both domestic and imported emissions with a legend given in the top right. The color of each bubble indicates the highest contributing sector from both domestic and imported emissions for each country.

**Figure 3:**
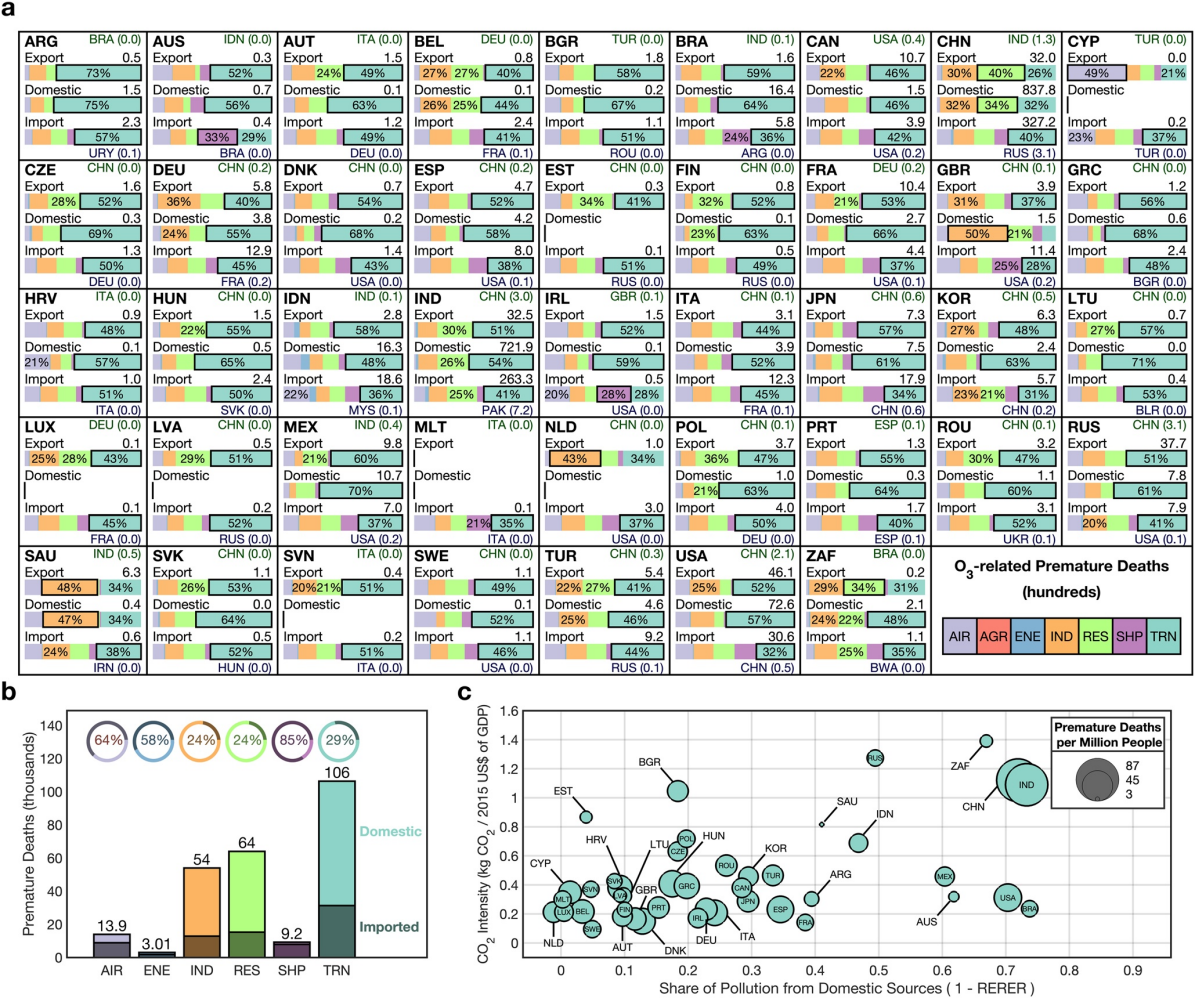
(a) Source contribution summaries of ozone (O_3_)‐related premature deaths in hundreds for each G20 country. See caption of Figure 2 for more details. Bars with small absolute contributions (<0.004) are excluded. (b) Total O_3_‐related premature death contributions in thousands from all major sectors. See caption of Figure 2 for more details; here, the agricultural sector was removed, and the air sector was included (c) bubble chart comparison of CO_2_ intensity and response to extra‐regional emission reductions for O_3_‐related per‐capita‐premature deaths in all G20 countries. See caption of Figure 2 for more details.

**Figure 4:**
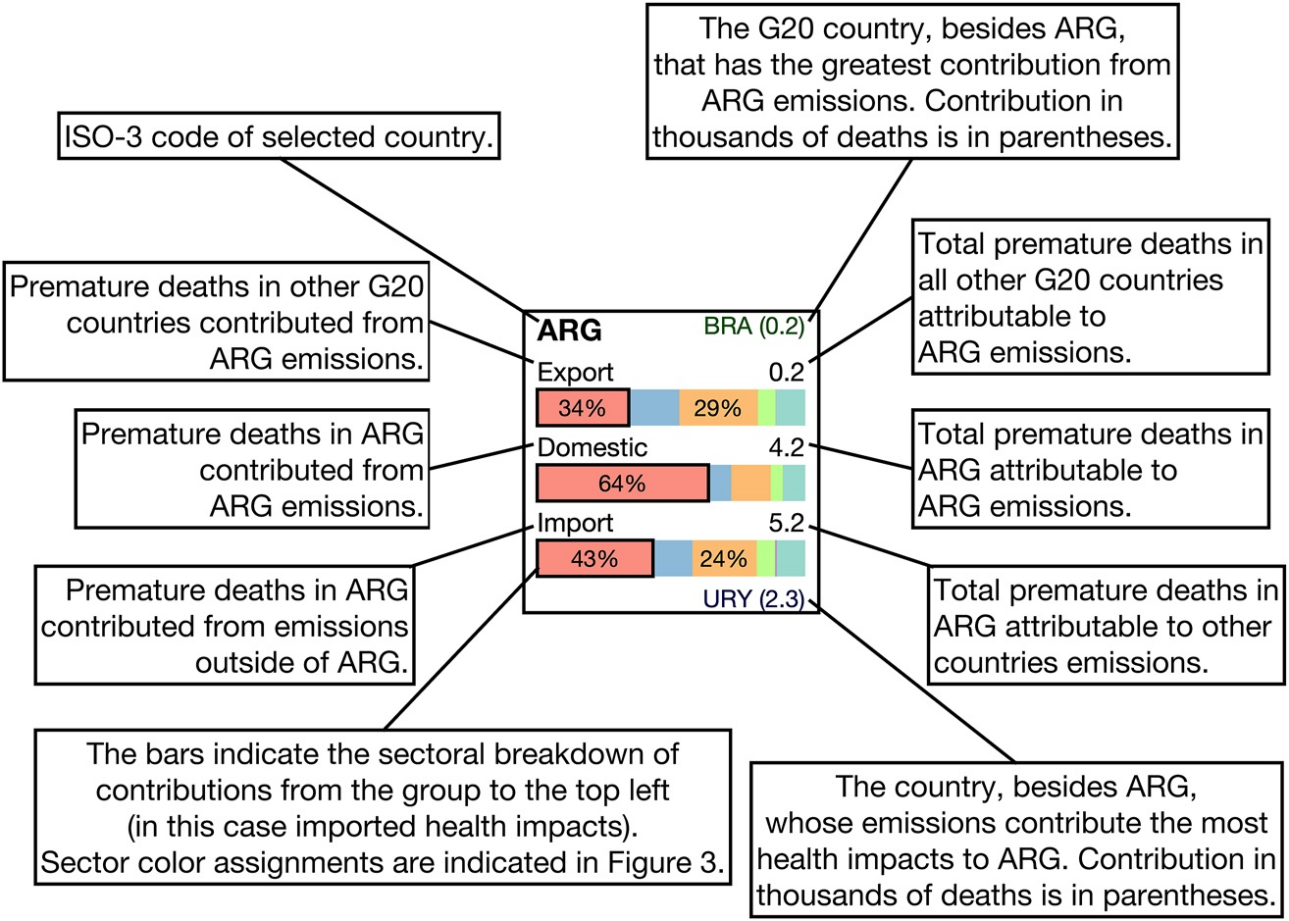
Explanatory diagram indicating how to interpret the results of Figures 2a and 3a.

**Figure 5:**
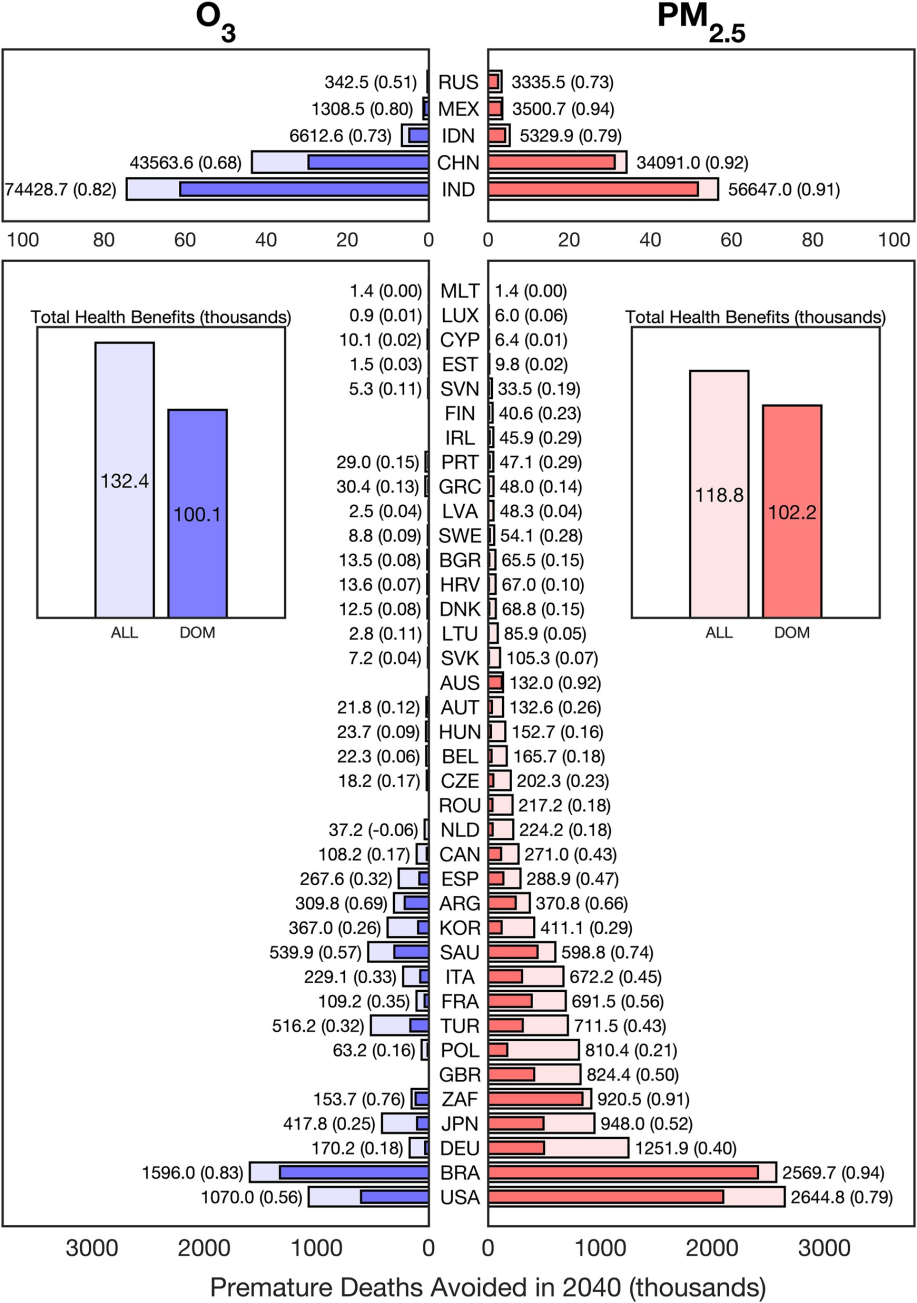
Projected premature deaths avoided in 2040 compared to ECLIPSE v5a current legislation (CLE) emissions from a 50% reduction in land transportation (TRN) emissions between 2020 and 2040. The “total health benefits” subplots indicate the premature deaths avoided in thousands for two reductions scenario, ALL and DOM. The ALL scenario considers the health benefits in a single country from all countries reducing their TRN emissions by 50% and the DOM scenario considers the health benefits in a single country if only that country reduced its TRN emissions by 50%. The five countries with the highest health benefits (India, China, Indonesia, Mexico, and Russia) are included at a different scale than the rest of the countries. The numbers at the end of each of the bars indicate the additional benefits afforded by the global emissions reduction and the number in the parenthesis indicates the ratio of benefits achieved from DOM compared to the benefits achieved from ALL.

**Figure 6:**
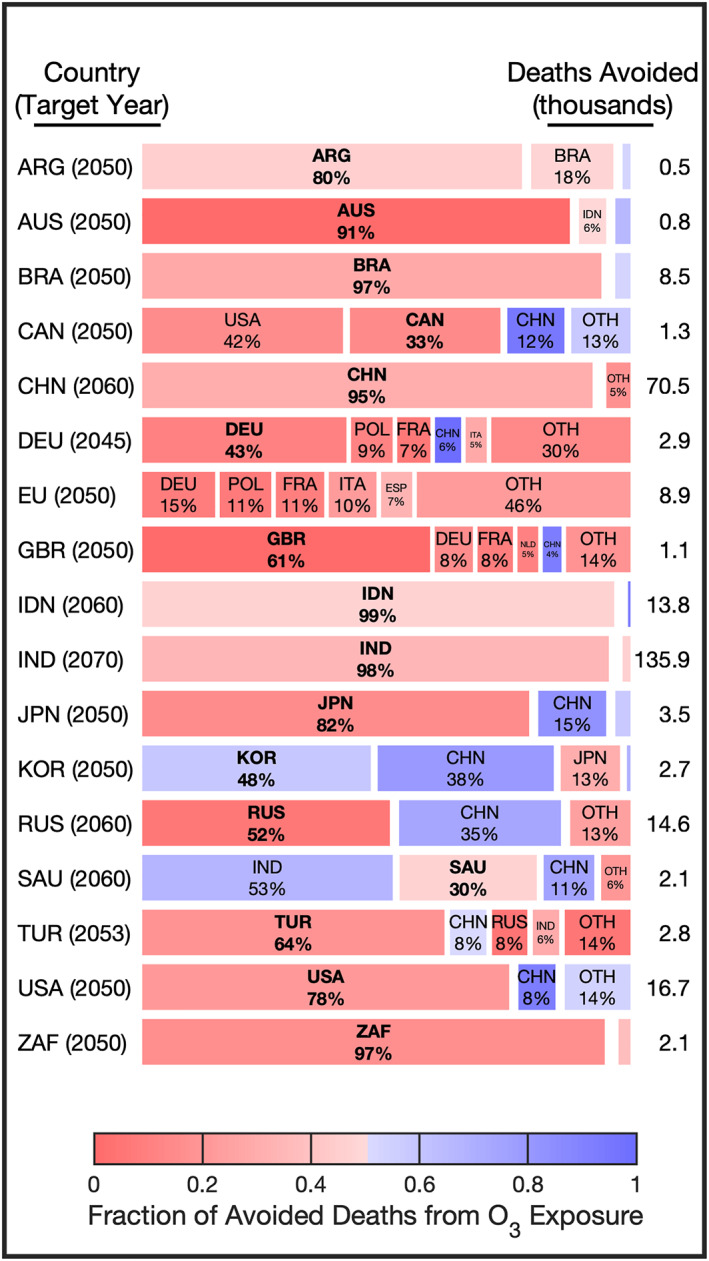
Co‐benefits of net‐zero CO_2_ targets in 2040. On the *y*‐axis we consider emission reductions in the 17 different G20 countries that have set net‐zero target CO_2_ years, labeled on the left side with the nation and target year. In the *x* direction, the blocks indicate the countries that would achieve the most health benefits from the emission action and the proportion of benefits. The color of a block indicates the amount of all co‐benefits that are attributable to reductions in ozone (O_3_) exposure and is equivalent to the O_3_ exposure reduction co‐benefits divided by the total co‐benefits from both O_3_ and fine particulate matter (PM_2.5_) exposure reductions. Redder colors indicate that the largest component of co‐benefits is attributable to reductions in PM_2.5_, bluer colors indicate that the largest component of co‐benefits is attributable to O_3_, and whiter colors indicate that co‐benefits are equally attributable to reductions in PM_2.5_ and O_3_.

The results in this work are separated into four major sections. First, we present the performance of the novel second‐order adjustment to the contribution calculation compared to finite difference tests from the forward model (Figure 1). Then, we exclusively consider source attribution from the perspective of regionality by characterizing the competing influences of domestic and upwind emissions on air pollution‐related health impacts in each G20 country (Table 1). Next, we discuss the additional complexity introduced by the inclusion of sectors in our source attribution allowing us to better examine dynamic relationships involving the transport of air pollution‐related premature deaths among G20 countries (Figures 2 and 3). Lastly, we assess the health benefits associated with two emission reduction scenarios: a 50% reduction in transportation emissions (Figures 4 and 5) and energy generation emission reductions associated with G20 countries staying on pace with their CO_2_ net‐zero target years (Figure 6).

**Table 1:** Summary of Imported and Domestic Premature Deaths Along With Response to Extra‐Regional Emission Reductions (the Fraction of Air Pollution‐Related Premature Deaths in a Country That Comes From External Sources) Values From All Sectors for All G20 Countries

Country	ISO‐3	Imported deaths (thousands)		Domestic deaths (thousands)		RERER	
		PM_2.5_	O_3_	PM_2.5_	O_3_	PM_2.5_	O_3_
Argentina	ARG	5.20	0.23	4.18	0.15	0.55	0.61
Australia	AUS	0.40	0.04	1.36	0.07	0.23	0.38
Austria	AUT	2.92	0.12	0.68	0.01	0.81	0.90
Belgium	BEL	4.30	0.24	0.47	0.01	0.90	0.97
Bulgaria	BGR	7.34	0.11	2.49	0.02	0.75	0.82
Brazil	BRA	4.39	0.58	30.29	1.64	0.13	0.26
Canada	CAN	3.94	0.39	4.26	0.15	0.48	0.72
China	CHN	67.03	32.72	821.28	83.78	0.08	0.28
Cyprus	CYP	0.43	0.02	0.01	0.00	0.99	0.99
Czech Republic	CZE	5.34	0.13	1.62	0.03	0.77	0.82
Germany	DEU	25.64	1.29	13.60	0.38	0.65	0.77
Denmark	DNK	1.74	0.14	0.31	0.02	0.85	0.87
Spain	ESP	7.73	0.80	3.86	0.42	0.67	0.65
Estonia	EST	0.57	0.01	0.07	0.00	0.90	0.96
Finland	FIN	1.25	0.05	0.59	0.01	0.68	0.90
France	FRA	8.45	0.44	9.25	0.27	0.48	0.62
United Kingdom	GBR	11.45	1.14	8.16	0.15	0.58	0.88
Greece	GRC	5.63	0.24	1.92	0.06	0.75	0.80
Croatia	HRV	2.74	0.10	0.35	0.01	0.89	0.91
Hungary	HUN	6.62	0.24	1.57	0.05	0.81	0.83
Indonesia	IDN	24.17	1.86	26.61	1.63	0.48	0.53
India	IND	86.87	26.33	350.95	72.19	0.20	0.27
Ireland	IRL	0.52	0.05	0.09	0.01	0.85	0.78
Italy	ITA	17.17	1.23	9.39	0.39	0.65	0.76
Japan	JPN	24.58	1.79	12.27	0.75	0.67	0.71
South Korea	KOR	10.62	0.57	2.01	0.24	0.84	0.71
Lithuania	LTU	2.41	0.04	0.20	0.00	0.92	0.90
Luxembourg	LUX	0.17	0.01	0.00	0.00	0.99	1.00
Latvia	LVA	1.64	0.02	0.13	0.00	0.92	0.91
Mexico	MEX	2.74	0.70	11.97	1.07	0.19	0.40
Malta	MLT	0.20	0.01	0.00	0.00	1.00	1.00
Netherlands	NLD	5.16	0.30	0.87	0.00	0.86	1.01
Poland	POL	14.35	0.40	9.18	0.10	0.61	0.80
Portugal	PRT	2.50	0.17	0.57	0.03	0.81	0.85
Romania	ROU	12.92	0.31	5.92	0.11	0.69	0.74
Russia	RUS	49.75	0.79	82.08	0.78	0.38	0.51
Saudi Arabia	SAU	3.72	0.06	2.50	0.04	0.60	0.59
Slovakia	SVK	3.14	0.05	0.43	0.00	0.88	0.92
Slovenia	SVN	0.70	0.02	0.12	0.00	0.85	0.95
Sweden	SWE	2.16	0.11	0.70	0.01	0.75	0.95
Turkey	TUR	13.93	0.92	13.09	0.46	0.52	0.67
USA	USA	19.02	3.06	79.83	7.26	0.19	0.30
South Africa	ZAF	1.45	0.11	12.63	0.21	0.10	0.33

RERER	0.00	0.10	0.20	0.30	0.40	0.50	0.60	0.70	0.80	0.90	1.00
O_3_‐related deaths (thousands)	0.0	0.5	1.0	1.5	2.0	2.5	3.0	3.5	4.0	4.5	>5.0
PM_2.5_‐related deaths (thousands)	0	5	10	15	20	25	30	35	40	45	>50

*Note.* Rows are color coded based on the magnitude of the cell values indicated by the legend at the bottom of the table.

Before presenting these results, we define a few key terms here. By “deaths” we mean the premature deaths statistically associated with long‐term exposure to PM_2.5_ or O_3_. We use “source” to refer to a country where emissions came from and “receptor” to refer to a country where health impacts from PM_2.5_ and O_3_ exposure occur. Considering any country, we define “imported” health impacts as deaths in that country that originates from emissions occurring outside of that country. “Domestic” health impacts in any country are defined as deaths in that country originating from emissions within that same country. “Exported” health impacts of a country refer to emissions that originate within a country but result in deaths in other G20 countries, not in that country. Lastly, we define the metric “RERER,” or the response to extra‐regional emission reductions, to be the fraction of a health impacts in a country that comes from external sources; it is defined as “imported” health impacts divided by “net” health impacts (“imported” plus “domestic” health impacts).

We will refer to all countries in the results section using their ISO‐3 codes as opposed to their country names. The ISO‐3 codes are listed in Table 1 for the G20 nations; all other nations ISO‐3 codes are included in the Supporting Information S1.

### 3.1 Second‐Order Contribution Calculation Performance

To determine the effectiveness of the second‐order contribution calculation compared to the standard first order calculation, we performed finite difference tests in 21 grid cells in which we both increased and decreased NO_x_ emissions by 50% and calculated the impact on O_3_ exposures in each of the G20 countries. We then consider the country that had the strongest response for each test and compared the response in the forward model test with the adjoint projection from the same emission perturbation using both the first‐order and second‐order contribution calculation (Figure 1). Overall, the second‐order approach had smaller normalized mean biases of +1.2% and −18.0%, when considering perturbations in both the positive and negative direction, than the first‐order approach with biases of +11.6% and −30.8%, respectively. This improvement in bias came at the cost of a slight degradation in correlation; the second‐order approach had *R* ^2^ values of 0.92 and 0.86 compared to the first‐order approach with 0.96 and 0.96.

### 3.2 Regional Source Apportionment

To characterize the regional sources of PM_2.5_‐ and O_3_‐related premature deaths in G20 countries we consider the imported and domestic deaths in each country and calculate the RERER values associated with these estimates (Table 1). First, we explore the absolute imported and domestic premature deaths, then we consider differences in the RERER values between O_3_ and PM_2.5_, and lastly, we describe patterns in the RERER values for PM_2.5_. We denote RERER values for each of the two pollutants using subscripts: RERER_PM2.5_ and RERER_O3_.

We first consider the influence of imported and domestic emissions on health impacts in an absolute sense. Across all countries, absolute imported and domestic premature deaths from PM_2.5_ exposure are higher than deaths from O_3_ exposure. Unsurprisingly, the most populous G20 countries bear the most domestic air pollution‐related health impacts; the location of a country and its upwind neighbors are irrelevant to its domestic contribution. Owing to this, these contributions largely mirror population and emissions data. To elaborate, there are three G20 countries that make up a majority of all domestic G20 premature deaths, China (CHN), India (IND), and the United States (USA). Together, these countries contributed 82% and 95% of all domestic PM_2.5_‐ and O_3_‐related premature deaths in G20 countries, respectively, and they also contained 64% of the total G20 population and emitted 58% of all NOx emissions from G20 countries in 2010.

There is not as clear of a dependency on population and emissions data when considering imported PM_2.5_‐ and O_3_‐related premature deaths. While population is still a key characteristic of imported health impacts, the location of a country and its proximity to high emitters can be more important. Despite it being the ninth most populous G20 country, Germany (DEU) has the fourth most imported PM_2.5_‐ and O_3_‐related premature deaths of 25,643 and 1,293, respectively. Germany has more imported premature deaths from both PM_2.5_ and O_3_ (26,936) than much more populous nations like Indonesia (IDN) (26,029), the United States (22,077), and Brazil (BRA) (4,970). In general, for both domestic and imported premature deaths, variability in baseline mortality rates across all six considered health outcomes can also explain some of the regional differences in absolute health impacts.

When comparing O_3_ and PM_2.5_ RERER values it is clear there are differences in how domestic and imported emissions contribute to the health impacts of these pollutants. Across all G20 countries, the average RERER value for O_3_, 0.73, is higher than that of PM_2.5_, 0.65, indicating that emissions from outside of a country have a greater impact on premature deaths from O_3_ than PM_2.5_ in general. This is not surprising given the longer lifetime of O_3_ compared to PM_2.5_, and it complements previous work (Anenberg et al., 2009) that found that reducing O_3_ precursor emissions in North America and Europe would result in greater benefits outside of these source regions than within. To assess the effectiveness of cooperative action in a number of countries, O_3_ exposure may be a better metric than PM_2.5_ exposure. There are notable exceptions to this pattern in RERER values for Ireland (IRL) and South Korea (KOR), which both have smaller O_3_ RERER values than PM_2.5_; for these countries, imported agricultural emissions, which only include chemical precursors of PM_2.5_, make up large components of their overall PM_2.5_‐related premature deaths at 25% and 36%, respectively. Because agricultural emissions do not contribute to O_3_, in our analysis, this imported source is not considered, which overall lowers imported premature deaths from O_3_ exposure. Regardless, for all countries, PM_2.5_ exposure reductions will still be responsible for greater health benefits from domestic emission reductions, and may be greater for foreign emissions reductions, due to the stronger relationship between exposure and increased relative risk of premature death for PM_2.5_ than O_3_. The trade‐off between transport and potency for the impacts of foreign sources of O_3_ versus PM_2.5_ is demonstrated in two policy applications presented in Section 3.4.

Countries with low RERER_PM2.5_ values (defined to be <0.3) exhibit at least one of three qualities: they are among the highest emitting countries, they have large surface areas, or they have few or no upwind neighbors that are high emitting countries. We use NO_x_ emissions as a metric to identify heavy polluters and find that on average low RERER_PM2.5_ countries emit 0.44 Tg of NO_x_ per year led by China, the United States, and India, which in 2010 emitted 6.9, 6.0, and 5.1 Tg of NO_x_, respectively. Low RERER_PM2.5_ countries also have large surface areas; on average they have areas of 0.86 million km^2^ led by the United States, China, and Brazil which have areas of 9.5, 9.4, and 8.6 million km^2^, respectively. Lastly, South Africa (ZAF), Indonesia, and Australia (AUS) are all examples of countries that are not directly downwind of heavy polluters and are geographically isolated. PM_2.5_‐related premature deaths are dominated by domestic sources in these low RERER_PM2.5_ countries; consequently, these countries have more control over their air pollution and its associated health burden.

High RERER_PM2.5_ countries (>0.6) are, in contrast, often characterized by smaller emissions and geographic size. On average they emit 0.02 Tg of NOx per year led by Japan (JPN), South Korea, and Germany which in 2010 emitted 1.1, 0.5, and 0.5 Tg, respectively. High RERER countries on average have areas of 0.02 million km^2^ led by Spain (ESP), Japan, and Sweden (SWE) which have the same area of 0.5 million km^2^. Examples of countries directly downwind of heavy polluters include Japan, South Korea, Lithuania, and Latvia. Imported emissions are the dominant source of air pollution health impacts in these high RERER_PM2.5_ countries outweighing the impact of their own domestic emissions. In contrast to low RERER_PM2.5_ countries, domestic action is less effective and addressing air pollution requires a greater dependence on collective action including reductions from both domestic and imported sources to improve air quality and reduce air pollution‐related health impacts.

Lastly, the countries with intermediate RERER_PM2.5_ values (>0.3 and <0.6) are often characterized by some combination of the traits of the two prior groups. To take one example, the United Kingdom (GBR) is a heavy polluter with 0.5 Tg of NOx emitted in 2010 but has a relatively small area of 0.29 million km^2^ and is not directly downwind of any heavy polluters. Collective action is needed in these countries to improve their air quality as well but less so than the high RERER_PM2.5_ countries. These trends observed in RERER_PM2.5_ values are also applicable to RERER_O3_.

### 3.3 Sectoral Source Apportionment

By considering the influence of sectoral sources in conjunction with regional sources it becomes possible to trace clear patterns and narratives of the flow of pollutants from their origins to their health endpoints. This added detail is evident in the breakdowns presented for PM_2.5_‐ (Figure 2a) and O_3_‐related (Figure 3a) premature deaths and in the associated explanatory diagram (Figure 4). The two cases discussed in the following two paragraphs cover just a brief amount of the content available; however, they are intended to serve as examples of how analysis could be performed from these results and the more detailed results available in data set associated with this work.

For example, we first consider source attribution from the perspective of a single source: agricultural emissions from China. This source is a dominant contributor of domestic PM_2.5_‐related health impacts in China; it is responsible for 52% of the country's 0.82 million domestic premature deaths. The PM_2.5_‐related health impacts associated with agricultural emissions from China are not exclusively realized domestically; South Korea imports 10.6 thousand PM_2.5_‐related premature deaths from all countries and 32% of these deaths originate from agricultural emissions from China. This is nearly double the second leading imported source China PM_2.5_‐related premature deaths in South Korea: the 14% contributed from industrial emissions from CHN. In fact, imported agricultural emissions from China are responsible for 1.7 times as many PM_2.5_‐related premature deaths in South Korea than all domestic sources combined. The next closest downwind G20 country from China, Japan, also imports many PM_2.5_‐related premature deaths from China; 12.9 thousand deaths in Japan originate from emissions from China which is slightly more than the 12.3 thousand deaths that originate from domestic emissions in Japan. Imported deaths in Japan have a different source profile than South Korea; surprisingly, agricultural emissions from China only make up 11% of the 24.6 thousand PM_2.5_‐related premature deaths imported from all countries. Both IND and RES emissions from China contribute larger shares of imported PM_2.5_‐related premature deaths in Japan at 19% and 13%, respectively. Ultimately, a dominant domestic sectoral contributor to PM_2.5_‐related premature deaths, or even a sectoral contributor that extends to directly downwind neighbors will not necessarily be the dominant contributor to further downwind receptors due to differences in the chemical species emitted from different sectors. The importance of the role of NH_3_, especially from agricultural emissions, on the formation of PM_2.5_ in China is well‐documented (Cohan et al., 2005; Wu et al., 2016); however, decreasing agricultural emissions could come with the trade‐off of an increase in acid deposition (Liu et al., 2019). Regardless, it is useful for countries to be able to identify both the regions and sectors that contribute the most to their pollution‐related health burden to effectively develop pollutant mitigation policies.

The previous discussion of agricultural emissions from China focused on the role of a single source and its influence on pollution‐related health impacts; however, our methodology is also capable of receptor focused analysis. In Germany, nearly two‐thirds of PM_2.5_‐related premature deaths originate from imported emissions. The location of Germany, downwind of many high emitting countries in Western Europe, naturally makes it a likely receptor of foreign sourced pollution‐related health impacts. While a majority of PM_2.5_‐related premature deaths in Germany originate from upwind emissions, this is not to say that emissions from Germany have few health consequences; Germany contributes 14.7 thousand PM_2.5_‐related premature deaths across all of its downwind G20 neighbors, more than the 13.6 thousand it contributes to itself. Over three quarters of all exported PM_2.5_‐related premature deaths from Germany originate from its agricultural, transportation, and energy generation emissions which contribute 32%, 28%, and 17%, respectively. There are nearly as many PM_2.5_‐related premature deaths in Germany that originate from imported emissions (25.6 thousand) as there are from domestic and exported emissions combined (28.3 thousand); these imported deaths are attributable to a number of sources. To name a few of the largest sectoral‐country sources, 1.1, 1.0, and 1.7 thousand PM_2.5_‐related premature deaths in Germany are attributable to agricultural, residential, and transportation emissions from France (FRA); 0.8 and 0.6 thousand deaths in Germany originate from agricultural and residential emissions from Poland (POL); 0.7 and 0.4 thousand deaths in Germany originate from transportation and agricultural emissions from Italy (ITA). A country like Germany, where PM_2.5_‐related premature deaths do not originate primarily from a single dominant source and are instead contributed by many smaller sources, presents the need for collective and comprehensive emissions mitigations in certain groups of G20 countries. A number of studies (Crippa et al., 2016; Liang et al., 2018; Viana et al., 2020; L. Wang et al., 2016) have highlighted the role of the long‐range transport of emissions on air quality in European countries along with more recent work (Spiteri & von Brockdorff, 2020) which has found a statistically significant relationship between transboundary emissions and premature death.

By aggregating across all countries, we estimate the overall domestic and imported PM_2.5_‐related premature deaths contributed from each sector (Figure 2b). We estimate that the agricultural sector contributed the most PM_2.5_‐related premature deaths across all G20 countries at 0.63 million premature deaths. A majority of these premature deaths are domestic agricultural contributions, with only 15% being attributable to imported emissions; this is less than other major sectors like the residential, industrial, transportation, and energy generation where 21%, 26%, 30%, and 35% of all PM_2.5_‐related premature deaths were imported. The large domestic contribution is partially attributable to the fact that most farming, and thus most agricultural NH_3_ emissions, occur in more inland regions of countries. Additionally, some of the largest countries like Russia (RUS), China, Brazil, and the United States are among the largest producers of agricultural emissions across G20 nations; generally, a larger country will produce more domestic health impacts than exported exactly because they are larger, and thus there is more time for secondary pollutants to form as they are advected across the country. For many countries, reducing emissions from the agricultural sector would be one of the most effective means to reduce domestic PM_2.5_‐related premature deaths; however, as mentioned previously, reductions in NH_3_ emissions could lead to an increase in acid deposition. To simultaneously mitigate acid deposition and air quality related health impacts, emissions of NO_x_ and SO_2_ (precursors of nitric and sulfuric acid) would need to be decreased in tandem. The four previously mentioned sectors, residential, industrial, transportation, and energy generation, make up a majority of imported PM_2.5_‐related premature deaths at 19%, 22%, 18%, and 15%, respectively. Overall, we estimate that anthropogenic emissions from all sectors contributed 2.00 million PM_2.5_‐related premature deaths in G20 countries and that 24% of these premature deaths originated from imported sources.

We next compare the RERER_PM2.5_ value of a country to the CO_2_ intensity of a country defined as the amount of CO_2_ emitted relative to the GDP of a country (The World Bank, 2020) (Figure 2c). Overall, countries with high RERER_PM2.5_ values (>0.6) generally have smaller CO_2_ intensities: on average 0.36 compared to 0.61 in other countries. The inverse is not necessarily true, that is, countries with average or low RERER_PM2.5_ values do not always have larger CO_2_ intensities, for example, Brazil (0.24), Australia (0.32), and the United States (0.31) all have CO_2_ intensities less than the average of high RERER_PM2.5_ countries. Countries that reduce CO_2_ emissions, or that have less CO_2_ emissions to begin with, will naturally emit less air quality precursor emissions which would largely impact domestic pollution. This would reduce the domestic fraction of PM_2.5_‐related health impacts in these countries and subsequently more pollution there would come from imported sources increasing the RERER_PM2.5_ value.

Lastly, we consider the sources of O_3_‐related premature deaths (Figures 3a–3c) in comparison to the PM_2.5_ results discussed in the previous few paragraphs. Primarily, there are two clear patterns that distinguish O_3_‐related health impacts from PM_2.5_: the dominance of the transportation sector and the increased impact of imported sources. Across all G20 countries, transportation emissions contributed 42% of the 0.25 million O_3_‐related premature deaths or 0.11 million O_3_‐related premature deaths. In a relative sense, transportation emissions were more important to the O_3_‐related health burden than to the PM_2.5_‐related health burden where transportation emissions contributed 11% of the 2.00 million PM_2.5_‐related premature deaths or 0.23 million premature deaths. This is despite the stronger association between PM_2.5_ exposure and risk of premature death and attributable to the strong sensitivity of O_3_ to emissions of NO_x_. There are a few interesting exceptions to the dominance of the transportation sector: a majority of the domestic O_3_‐related health impacts of Malta (MLT) (64%) comes from the SHP sector while in Saudi Arabia (SAU), the industrial sector, which includes oil production, is the leading domestic source (47%). China and Ireland also have among the smallest percentages of their O_3_‐related premature coming from transportation emissions at 34% and 34% respectively. In all other countries transportation emissions make up 35% or more of all O_3_‐related premature deaths. Relationships between RERER_O3_ and CO_2_‐intensities are similar to PM_2.5_; countries with high RERER_O3_ values still tended to have lower CO_2_ intensities although there is slightly tighter clustering.

### 3.4 Estimating the Health Impacts of Specific Emission Scenarios

We next consider how the source attribution results presented above can be used to quantify the health impacts of sector‐specific interventions, through their application (see Methods Section 2.5) as a reduced form, off‐line, estimation tool, without rerunning an air quality model multiple times. Here, we consider two highly simplified interventions which, by targeting different sectors, show the range of importance of local versus external sources of O_3_ versus PM_2.5_. We first assess how a 50% reduction from all TRN emissions by 2040 would impact PM_2.5_ and O_3_‐related premature deaths in G20 countries (Figure 5) relative to ECLIPSE v5a CLE. In this scenario, we consider two distinct cases. In the first case, denoted as “DOM” and by solid blue and red bars in the figure, we consider the health benefits of this 50% reduction in a single country if only that G20 country applied this emissions reduction; this country is indicated by the label to the right or left of the bar for O_3_ and PM_2.5_, respectively. In the second case, denoted as “ALL” and by the light blue and red bars in the figure, we consider the premature deaths avoided in a single country if every country in the world applied this emissions reduction.

Overall, a 50% reduction in TRN emissions by 2040 would result in 251 thousand PM_2.5_‐ and O_3_‐related premature deaths avoided in the ALL case and 202 thousand PM_2.5_‐ and O_3_‐related premature deaths avoided in the DOM case relative to current legislation. In both cases, a 50% reduction in TRN emissions would result in overall health benefits almost equally split between reductions in PM_2.5_ and O_3_ exposure; O_3_ exposure reductions would be responsible for 53% of the deaths avoided in the ALL case and 49% of the deaths avoided in the DOM case. This result includes proportionally higher O_3_‐related premature deaths avoided than other studies which project the impact of TRN emission controls to future years (Anenberg et al., 2017). This is due to high estimates of O_3_‐related premature deaths avoided in some of the most populous G20 nations including India, China, and Indonesia; in most other countries O_3_‐related premature deaths avoided are substantially smaller than PM_2.5_‐related premature deaths avoided. While exposure to PM_2.5_ is responsible for more premature deaths in G20 countries in total, O_3_ exposure is generally more sensitive to emissions from the TRN sector, which are primarily of NO_x_, than PM_2.5_ at the country level. This is not globally true; in some areas in some countries that is, China and India, PM_2.5_ and O_3_ exposures have similar sensitivities to TRN emissions and at the local and city level the relationship between pollutant exposures and NO_x_ emissions is far more complex. In the ALL case, O_3_ health benefits are actually greater than PM_2.5_ health benefits, by ∼10%. The number of premature deaths avoided from foreign action, ALL–DOM, is nearly double for O_3_‐related premature deaths (32.3 thousand) than PM_2.5_‐related premature deaths (16.6 thousand) driven primarily by China and India whereas for most other countries the opposite is generally the case. This scenario indicates that despite PM_2.5_ exposure having a stronger association with premature death, both the source sector and region play an integral role in determining the pollutant that drives the overall long‐range transport health impacts or benefits.

On an individual country level, there is variability in both the pollutant responsible for more overall health benefits and most premature deaths avoided from foreign emission reductions. For most countries, PM_2.5_ health benefits outweigh O_3_ health benefits; however, among the most populous G20 nations that would have the largest health benefits from a 50% reduction of TRN emissions, this is not true. For example, India and China both have 30% more total O_3_‐attributable health benefits than PM_2.5_‐attributable health benefits. In most of the countries which have the greatest estimated health benefits from this scenario, a majority of the benefits could be achieved by domestic action; however, there are a few exceptions to this rule. For PM_2.5_‐health benefits countries that more than double their benefits by cooperative action in the ALL case relative to the DOM case include Turkey (TUR) (43% domestic), South Korea (29%), Germany (40%), and Poland (21%). In less populous nations, where health benefits from emission reductions are smaller, collective action in the ALL case can be much more beneficial than domestic action. For O_3_‐attributable health benefits, there are additional populous nations with greater impacts from the ALL case; beyond Turkey (32%), South Korea (26%), Germany (18%), and Poland (16%), there is Japan (25%), Spain (ESP) (32%), and Italy (33%) again supporting the idea that the location and policy scenario of an emissions reduction play a role in determining the leading pollutant, O_3_ or PM_2.5_, responsible for long‐range health benefits.

In the final set of scenarios, we estimate the co‐benefits of reductions in CO_2_ emissions associated with countries staying on pace to achieve their net‐zero target years (Figure 6). These co‐benefits are, specifically, deaths avoided due to improvements in air quality from reductions in the precursor emissions of PM_2.5_ and O_3_. We limit this analysis to emission reductions from the energy generation sector and assume that reductions in air pollutant precursor emissions would correspond linearly with CO_2_ reductions and that CO_2_ reductions would occur linearly through time toward zero emissions in net‐zero target years. Additionally in this simple scenario we do not consider the additional air quality precursor emissions that would be emitted from the cleaner energy sources. These are assumptions that greatly simplify the complexity of CO_2_ emission reductions, but the added simplicity allows for an approximate estimate of CO_2_‐emission reduction co‐benefits. The framework developed in this study could be extended beyond the simple examples provided to more complex emission scenario analyses.

Of all the G20 nations that have set a net‐zero target year, India has the latest date set for 2070; despite this, we estimate that IND staying on pace with its target would avoid 136 thousand PM_2.5_‐ and O_3_‐related premature deaths in 2040. This is much more than the 71 thousand and 17 thousand deaths avoided from action by China and the United States with net‐zero target years of 2060 and 2050, respectively. In India, China, and the United States, a majority of the co‐benefits associated with decarbonization would occur domestically at 98%, 95%, and 78%, respectively. While this is as well the case in most countries, there are a few exceptions: due to emission reductions in Canada (CAN), 42% of all premature deaths avoided occur in the United States compared to 33% in Canada, and due to emission reductions in Saudi Arabia, 53% of all premature deaths avoided occur in India compared to 30% in Saudi Arabia. The co‐benefits in the United States from Canadian CO_2_ emission reductions are primarily attributable to PM_2.5_ exposure emission reductions; this is due to the proximity of cities in the United States, mostly in the northeast, to large energy generation units in Canada. For Saudi Arabian CO_2_ emissions reductions, a majority of co‐benefits in India are attributable to O_3_ exposure reductions; a majority, 53%, of the direct downwind benefits, occurring domestically, are attributable to reductions in PM_2.5_ exposure while benefits in downwind nations that are further away are mostly attributable to O_3_ exposure reductions at 68% in India and 76% in China. We consider the co‐benefits of decarbonization in Germany separately from the EU because Germany has set its own earlier net‐zero target year. We estimate that emission reductions in EU countries, with the exception of Germany, would result in 1.3 thousand deaths avoided in Germany compared to the 1.2 thousand deaths that would be avoided from decarbonization in Germany despite an earlier net‐zero target year.

In interpreting these results, it is important to note that we only assess co‐benefits in G20 countries; the health co‐benefits of CO_2_ reductions would be much larger than indicated here if health benefits in other countries outside of the G20 were assessed. The health co‐benefits associated with the emission reduction in our study are substantially smaller than estimates of total avoidable deaths from fossil fuel emissions (Lelieveld et al., 2019) as estimated by another study; however, this is attributable to a few elements of this other study including the consideration of additional causes of death (Burnett et al., 2018), consideration of other sectors beyond energy generation, and the estimate of benefits in all countries as opposed to just the G20. The results presented for the prior scenarios consider changes in population, baseline disease rates, and age demographics in 2040 relative to 2010 values. To assess how impactful these changes are, we additionally calculate the total mortality, the product of population and baseline disease rates, in both 2010 and 2040. Overall, we estimate that total mortality from the outcomes considered in this study are 2.9 times greater in 2040 than in 2010 across all G20 countries. The total mortality values in 2040 relative to 2010 ranged from 1.1 times greater in Bulgaria to 5.8 times greater in South Korea. When interpreting the results from the emission scenarios, the influence of demographic changes, primarily on the overall magnitude of health impacts but also, to a lesser extent, on country‐by‐country variability should be considered.

### 3.5 Uncertainty Analysis and Limitations

In this section, we briefly discuss uncertainty and limitations of our work in a qualitative sense; however, in Section 3.1, and in Supporting Information S1, we present a quantitative discussion of uncertainty in more detail. Bottom‐up emissions inventories, like both the ECLIPSE and HTAP inventories, make assumptions regarding emissions factors and activities for specific sectors due to the inherent ambiguity involved in classifying sources at the global scale. It should also be noted that global inventories, like ECLIPSE and HTAP, can differ significantly from local emissions inventories in many areas of the world (Huneeus et al., 2020), especially outside of North America and Europe. While the emissions inventories undoubtedly introduce uncertainty into our analysis, one of the benefits of our approach is the capability to switch between emissions inventories, reducing this source of uncertainty as newer products are released. When we consider a 50% reduction in transportation emissions, we note that we do not account for any increase in electricity load and subsequent increases in associated emissions. The impact of this unaccounted‐for increase on air pollution is likely substantially smaller than a 50% reduction in transportation emissions; however, this does introduce uncertainty into our assessment.

All models make assumptions and simplifications that deviate from the complex natural systems they aim to represent; GEOS‐Chem has proven to capture air pollutant concentrations well compared to observations (Heald et al., 2012; Henze et al., 2009). Additionally, by including a satellite‐derived product with good agreement compared to observations (van Donkelaar et al., 2016) in the cost‐function for PM_2.5_, we correct for model biases. The adjoint calculation also introduces uncertainty into our analysis outside of the forward model simulation. The adjoint calculation only calculates the local‐linear sensitivity of a pollutant exposure to emissions; this sensitivity is representative for small emission changes but can become increasingly unreliable as larger emission changes are evaluated and non‐linear effects become more important. We partially account for this uncertainty by applying the second order approach for the O_3_ and NO_x_ relationship and by scaling first order sensitivities as discussed in greater detail in the Supporting Information S1. Uncertainty introduced in the health impact analysis has been shown to be a leading source of uncertainty in modeling set‐ups similar to the one used in this study (Nawaz et al., 2021). In our analysis we assume equal toxicity of all PM_2.5_ components as policies generally focus on total PM_2.5_ and because there is a larger body of research on the health impacts associated with total PM_2.5_ exposure compared to individual components.

The epidemiological studies that form the foundation of the relative risk exposure relationships used in our analysis have large uncertainties along with population data and baseline mortality rates. These uncertainties apply to all scenarios explored in this work so they should not affect the overall conclusions; however, uncertainties in the relative risk calculation would reflect changes in PM_2.5_ composition and concentrations. There are also differing means of calculating the health impacts from PM_2.5_ and O_3_ (Burnett et al., 2018; Jerrett et al., 2009; Murray et al., 2020; Turner et al., 2016). We calculate the uncertainty from the health impact assessment and include lower and upper bound values in the data set associated with this work.

## 4 Discussion and Conclusions

Characterizing the sources of pollutants like PM_2.5_ and O_3_ is undeniably complex; however, improving understanding is crucial to developing informed policy interventions. We present a framework for characterizing regional and sectoral sources of air pollution and for assessing the health benefits that could be achieved from reductions in emissions. The framework presented in this study is capable of identifying the regional and sectoral sources of air pollution in individual countries; in this work, we focused on the G20 countries as a case study and consider anthropogenic sources exclusively. Using the adjoint of GEOS‐Chem we calculated the sensitivity of PM_2.5_ and O_3_ exposures to their chemical precursors in each of the G20 countries. We also present a new approach which uses additional simulations to characterize a second order relationship between O_3_ and NO_x_ to account for the non‐linear formation of O_3_ in the sensitivities of O_3_ exposures to precursor emissions.

Uncertainty is introduced into our analysis from four major sources: the emissions data, the air quality model, the use of adjoint sensitivities for source attribution, and the health impact analysis. All of these sources of uncertainty are discussed in greater detail in Section 3.5 and in the Supporting Information S1. Keeping these sources of uncertainty in mind, we highlight some of the key findings of our analysis. The metric RERER is used to characterize the amount of PM_2.5_‐ and O_3_‐related premature deaths in each G20 country that comes from foreign emissions. Values of RERER depend on a number of factors such as the size of a country, how high of an emitter a country is, and the location of the country with respect to other high emitters. The PM_2.5_‐ and O_3_‐related premature deaths from domestic emissions were dependent exclusively on emissions magnitude, population, and mortality rates; however, health impacts from imported pollution were more location dependent. Among G20 nations, DEU has a relatively average population size, but it had among the highest imported PM_2.5_‐ and O_3_‐related premature deaths due to its proximity downwind from FRA, ITA, and other European countries. At the same time, emissions from Germany affect downwind neighbors like Poland; in both of these cases transportation emissions play a major role, which underscores the importance of supporting stringent EU‐wide policies such as the upcoming Euro VII standards that aim to reduce transportation NO_x_, although not necessarily to the same extent as the scenario considered in our analysis. One study (Anenberg et al., 2017) assessed the NO_x_ emission impacts of next generation vehicle standards across a similar group of countries and found reductions greater than 50% in 2040. Another study (Mulholland et al., 2022) estimated that between 2027 and 2050 NO_x_ emissions would reduce by 74% from Euro VI adoption and 93% with Euro VII standards.

The sectoral‐regional patterns identified in our analysis have clear policy implications. To avoid the most air pollution‐related premature deaths, China should prioritize reducing agricultural emissions; however, this should be done simultaneously with reductions in SO_2_ and NO_x_ to avoid increased acid deposition. Both South Korea and Japan have a stake in securing these emission reductions as many of their health impacts are attributable to imported agricultural emissions from China. Transportation emissions are a major contributor to O_3_‐related health impacts and are a major source of transboundary health impacts; this emphasizes the value of continued international cooperation to strengthen vehicle emission control policies.

Emission scenarios were combined with our adjoint calculations and health impact assessments to quantify the benefits of air pollution‐related deaths avoided in G20 countries from different actions. First, we considered a 50% reduction in TRN emissions for two cases: one a global emission reduction, and another a domestic emission reduction in each G20 country. Overall, PM_2.5_‐ and O_3_‐related premature deaths avoided from the proposed reductions would be similar in number despite PM_2.5_ exposure being more harmful to public health as evidenced by epidemiological studies. This is because O_3_ exposures have a higher sensitivity to TRN NO_x_ emissions than PM_2.5_. The competing influences of the longer lifetime of O_3_ and the higher relative risk of premature death associated with exposure to PM_2.5_ determine the predominant premature death causing pollutant associated with long‐range transport. Our analysis suggests that PM_2.5_ is not uniformly the predominant pollutant associated with trans‐boundary health impacts; considerations of both country and sector are needed to make this determination. We estimate that 251 thousand premature deaths would be avoided in G20 countries in 2040 if TRN emissions were reduced by 50% globally by 2040.

The second emission scenario we consider is a set of CO_2_ emission reductions attributable to achieving net‐zero CO_2_ targets. Many countries are not on pace to achieve net‐zero CO_2_ emissions by their target years; this slow action could be responsible for many excess deaths from additional air pollution exposures that would be reduced if emissions from energy generation units were reduced. We estimate that in 2040, 290 thousand premature deaths could be avoided if G20 countries achieved their targeted CO_2_ emission reduction progress, from energy generation, by 2040. Considering all net‐zero target years, a majority of these potential health benefits would be due to decarbonization in Indonesia despite it having the latest target year of all G20 nations with targets, 2070. This is attributable to high‐energy generation emissions in IND from its reliance on coal as well as its large and growing population. Many other countries and regions would have large domestic benefits from achieving their CO_2_ targets including China, the United States, Indonesia, Russia, Brazil, and the EU. One exception is Canada; it would only be the second‐largest benefactor of its CO_2_ emission reductions behind the United States highlighting the importance of regional cooperation to reduce emissions from energy generation.

The analysis presented in this study introduces an approach for characterizing the sensitivity of air pollution‐related health impacts to changes in emissions that can be used for both source apportionments and emission scenario assessments. Future work building off of this study could include the calculation of adjoint sensitivities for additional countries, expansion of this approach to other pollutants associated with health impacts, like nitrogen dioxide, health benefit assessments from more detailed emission scenarios, and the application of the second‐order contribution calculation to other non‐linear relationships between pollutants and precursor such as secondary inorganic and organic aerosol formation. As more detailed emissions inventories and satellite‐derived products become available the source apportionments and health impact assessments in this work should be refined.

## Conflict of Interest

The authors declare no conflicts of interest relevant to this study.

## Supporting information

Supporting Information S1

## Data Availability

All new data generated in this project is stored in the CU Scholar data repository which adheres to the common Enabling FAIR data Project (Nawaz et al., 2022). Newly generated data includes source apportionments of PM_2.5_‐ and O_3_‐related health impacts for each of the G20 receptor countries by sectoral and country source and projected 2040 health impacts associated with the emission scenarios discussed in this work. *Code Availability:* The codes developed for this study are available from the corresponding author upon reasonable request.
