# Supplementary material for: A Source Apportionment and Emission Scenario Assessment of PM2.5‐ and O3‐Related Health Impacts in G20 Countries
Source: Geohealth. 2023 Jan 4;7(1):e2022GH000713. doi: 10.1029/2022GH000713 (PMC9811479; doi:10.1029/2022GH000713)
Supplement: Supplementary file 1 — Supporting Information S1 [file GH2-7-e2022GH000713-s001.docx]

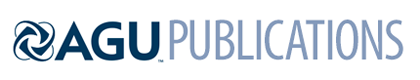


*GeoHealth*

**A source apportionment and emission scenario assessment of PM_2.5_- and O_3_-related health impacts in G20 countries**

M. Omar Nawaz*^1^, Daven K. Henze^1^, Susan C. Anenberg^2^, Caleb Braun^3^, Joshua Miller^4^, Erik Pronk^4^

^1^Department of Mechanical Engineering, University of Colorado Boulder, Boulder, CO, 80301, USA

*^2^George Washington University Milken Institute School of Public Health, Washington, DC, 20052, USA*

*^3^Climate Cabinet, San Francisco, CA, 94104*

*^4^The International Council on Clean Transportation, San Francisco, CA, 94105, USA*

**List of Contents**

S1.0 Uncertainty analysis 2

S1.1 Emissions inventories 3

S1.2 Air quality simulations 7

S1.3 Adjoint sensitivity analysis 8

S1.4 Health impact assessment 10
S1.5 Map of G20 member states 11

S1.6 ISO-3 code mapping 11

S1.7 References 16

**Introduction**

In this supporting information we present a detailed uncertainty analysis in sections S1.0 to S1.4, in S1.5 we present an explanatory diagram for Figures 2 and 3 of the main text, and a mapping of country names to ISO-3 codes in S1.6. Lastly, referenced works for the supplemental are included in S1.7. We present six figures in this supplemental: surface level emissions for HTAP (Figure S1), ECLIPSE (S2), and ECLIPSE projections (S3), a comparison of cost-function concentrations to observations (S4), a comparison of the adjoint model to finite difference forward model simulations (S5), and an explanatory diagram for Figures 2 and 3 of the main text (S6). Additionally, we include two tables: a comparison of HTAP and ECLIPSE emissions in G20 countries broken down by precursor species (Table S1) and a mapping of country names to ISO-3 codes for all countries we consider (Table S2).

**S1.0 Uncertainty analysis**

Throughout our study, we make assumptions and simplifications in order to make our analysis feasible; however, these assumptions and simplifications introduce uncertainty into our final results. In this discussion, we quantify, characterize, and compare uncertainties across the four major steps of our analysis: the emissions inventories, the forward model simulation of pollutant concentrations, the adjoint calculation of pollutant sensitivities, and the health impact assessment. We estimate how the simplifications we make at each of these steps contributes to uncertainty and we determine that the health impact assessment is likely the largest source of uncertainty in our analysis which is consistent with previous studies (Nawaz et al., 2021; Nawaz & Henze, 2020). Though uncertainty is also introduced by other components of our analysis like the satellite-derived data and meteorological inputs, these are excluded from our discussion as they are discussed in their associated works (Molod et al., 2012; van Donkelaar et al., 2016) and are comparatively smaller than uncertainty introduced in our analysis.

To present this uncertainty analysis in a consistent manner we first define a few key terms that are used throughout this supplement. We adopt the definitions of these terms from a previous study (Mallet & Sportisse, 2006) in which they define the “error” to be the difference between model outputs and observations and the “uncertainty” as the range of values in which the model outputs may lie with a high degree of confidence. Beyond these fundament terms we use a number of statistical terms to account and characterize error and uncertainty. The normalized mean bias (NMB) refers to the sum of the difference between predicted and observed values divided by the sum of observed values:

$$NMB= \frac{\sum_{i=1} \left( Y_{predicted}-Y_{observation} \right)}{\sum_{i=1} Y_{observation}}*100 (S1)$$

The sum of squared errors (SSE) is similar to the NMB; however, the normalization term is removed, and the numerator is squared:

$$SSE_{res}= \sum_{i=1} \left( Y_{predicted}-Y_{observation} \right)^{2} (S2)$$

The root mean square error (RMSE) is the square root of the sum of the difference between predicted and observed values divided by the total number of values:

$$RMSE=\sqrt{\sum_{i=1}^{N} \frac{Y_{predicted}-Y_{observation}}{N}} (S3)$$

Lastly, the coefficient of determination (R^2^) is the fraction of variation in the dependent variable that is predictable from the independent variable. It is one minus the ratio of the sum of squared errors in the residuals and the total sum of squares:

$SSE_{tot}= \sum_{i=1} \left( Y_{observed}-\bar{Y} \right)^{2} (S4)$

$R^{2}= 1-\frac{SSE_{res}}{SSE_{tot}} (S5)$

Where $\bar{Y}$ refers to the mean of observed values. These statistical terms are used to characterize uncertainty throughout our analysis. We do not combine the uncertainty ranges from all sources as we do not account for the co-variances between these distinct sources of uncertainty; instead, the lower and upper bound values presented in supplementals two and three are from the health impact assessment uncertainty exclusively.

­­­­­­

**S1.1 Emissions inventories**

Emissions are a key component of our analysis; we use them to drive the forward model simulation, to perform our source apportionment, and to assess the impacts associated with emissions projections. Given their fundamental role in our analysis, it is important to present total emission magnitudes and to quantify differences in inventories to characterize the country-level variability. As our results focus on anthropogenic emissions, we do not discuss uncertainty in natural emissions; however, this uncertainty, along with uncertainty in the anthropogenic emissions, contribute to differences between simulated concentrations and observations which is expanded upon in the next section.

Anthropogenic emissions from HTAPv2.2 (Janssens-Maenhout et al., 2015), for a base year of 2010, are used both as input for the forward model simulation and the source apportionment. Emissions from HTAPv2.2 are available at the 0.1° $\times$ 0.1° resolution. Spatial maps of HTAPv2.2 emissions, totaled across all sectors for each of the six key precursor species are included in Figure S1. A mosaic of different bottom-up inventories for different regions were used to develop HTAPv2.2; owing to this, each of these different regions have differing sources or uncertainty. In the HTAPv2.2 documentation, sectoral and species-specific uncertainties were considered in a qualitive sense for two aggregated groups: countries with and without well-maintained statistical infrastructure. They estimate that the highest uncertainties in emissions from HTAPv2.2 were from the transportation, residential, agricultural, and shipping sectors, and that emissions of NH_3_, BC, and OC were the most uncertain.


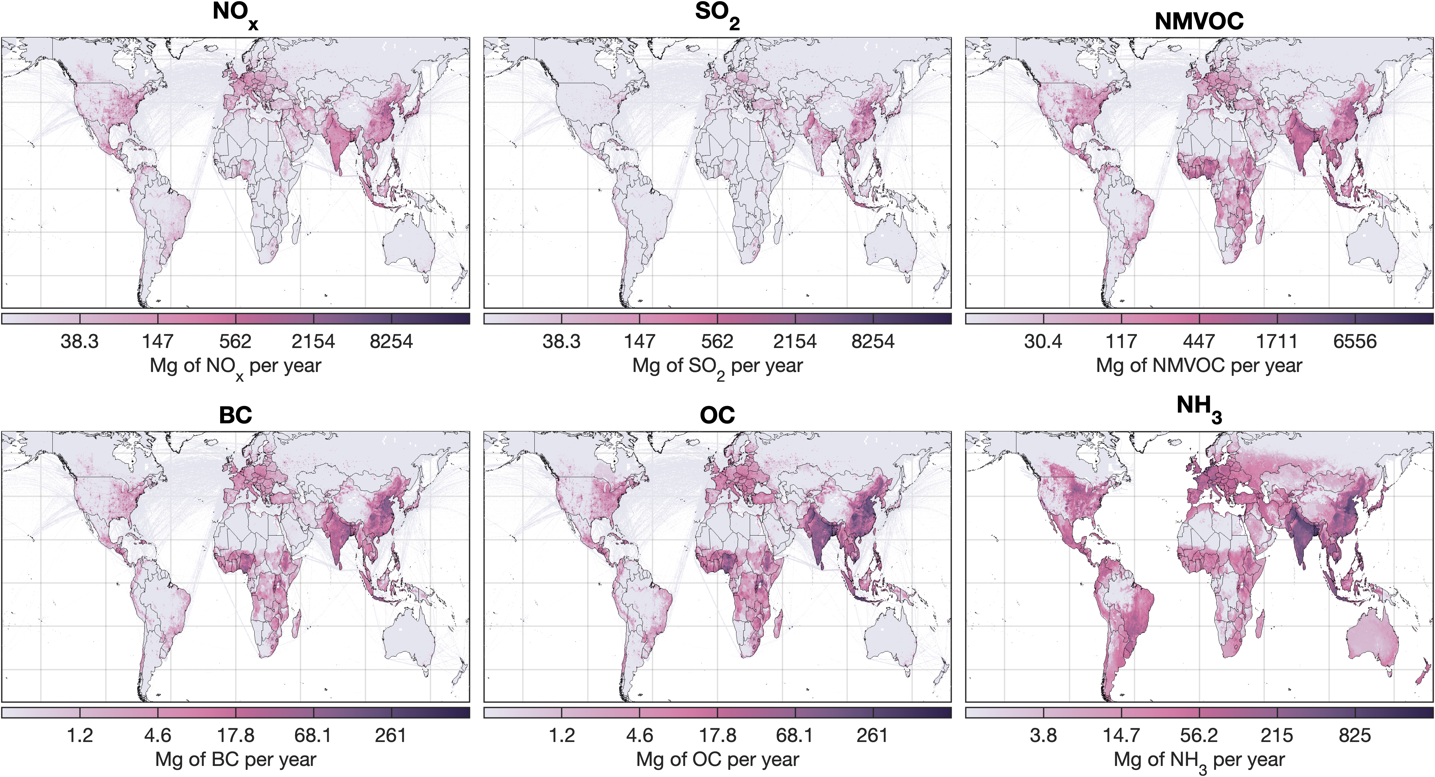


**Figure S1.** Spatial maps of annual anthropogenic emissions of the six major air quality precursor species from HTAPv2.2, aggregated across all sectors.

We use a different emission inventory, the baseline scenario (CLE) from ECLIPSEv5a (IIASA, 2021), for a base year of 2015, to perform our emission scenario impact assessment as this inventory includes projected emissions for future years. Emissions from ECLIPSEv5a are available at the 0.5° $\times$ 0.5° resolution. Uncertainties in ECLIPSEv5a are discussed in a qualitive sense in its documentation. In this analysis, they simulate the formation of PM_2.5_, O_3_, and other short-lived climate pollutants using multiple earth system and chemical transport models that are driven by ECLIPSEv5a emissions and compare these simulated pollutants with observations. By conducting these simulations, they found that there were underestimates of primary aerosol and aerosol precursors in parts of Russia and India, seasonal overestimates of SO_­2_ in Asia and Europe and overestimates of O_3_ in rural areas. Considering the same six precursors from Figure S1, in Figure S2 we present the surface level emissions from ECLIPSEv5a. In Figure S3 we present the absolute changes in ECLIPSEv5a for 2020, 2030, and 2040 compared to the baseline 2015 emissions for each precursor species.


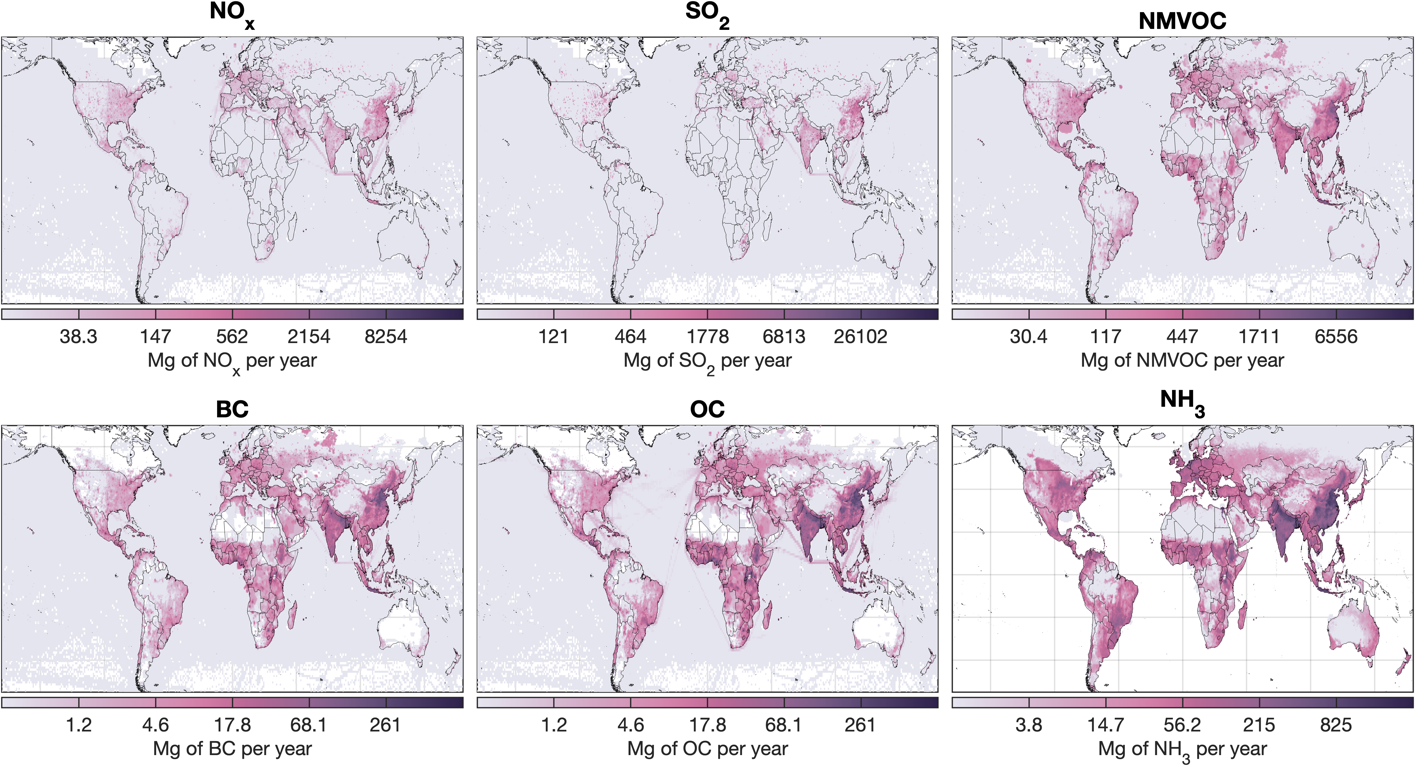


**Figure S2.** Spatial maps of annual anthropogenic emissions of the six major air quality precursor species from ECLIPSEv5a aggregated across all sectors.


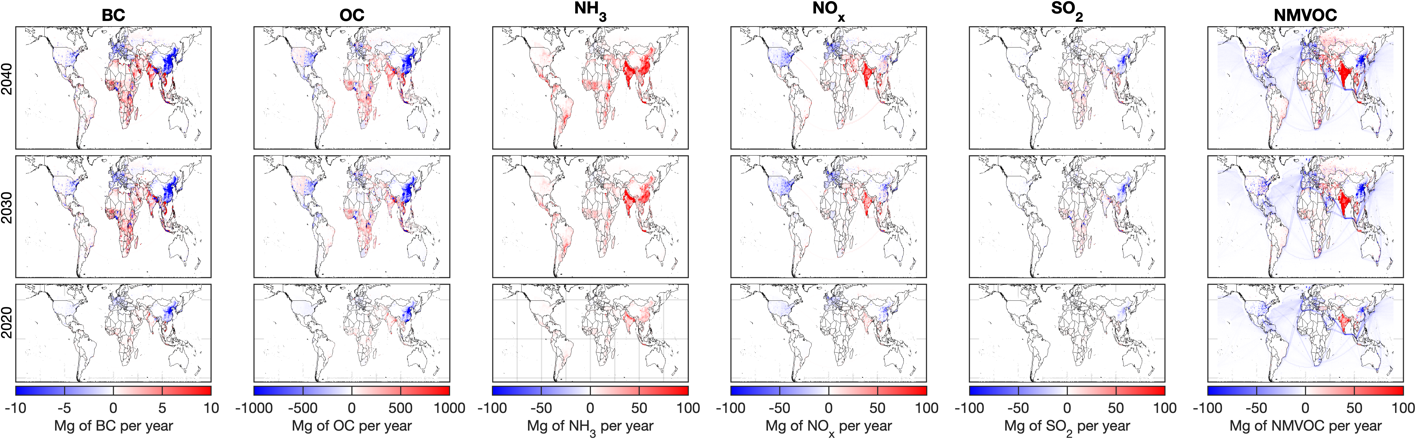


**Figure S3.** Absolute changes in emissions of the six precursor species from 2015 for 2020, 2030, and 2040.

To characterize uncertainties in a more quantitative manner we present the percent difference in emissions, in G20 countries, from both inventories separated by precursor species. Doing this allows us to quantify the relative variability in emissions values across inventories. In Table S1, we calculate the percent difference by species as:

$$Percent Difference=\frac{\left( ECLIPSE-HTAP \right)}{ECLIPSE}*100 (S6)$$

For most combinations of countries and species the percent difference between these two inventories is relatively small. The one major exception to this rule is MLT, which has larger percent differences due to its low overall emissions. Across all G20 countries, the median absolute percent differences were 28%, 41%, 13%, 24%, 39%, and 26% for BC, OC, NH_3_, NO_x_, SO_2_, and NMVOCs.

**Table S1.** Percent differences between 2015 ECLIPSE and 2010 HTAP emissions of the six precursor species for each G20 country

| **ISO3** | **BC** | **OC** | **NH_3_** | **NO_x_** | **SO_2_** | **NMVOC** |
| --- | --- | --- | --- | --- | --- | --- |
| ARG | +83% | +81% | +16% | +34% | -120% | -43% |
| BRA | +43% | +14% | +13% | +10% | -24% | -81% |
| AUS | +48% | +41% | +20% | -14% | -145% | -50% |
| CAN | +17% | -23% | +14% | -48% | +18% | -81% |
| MEX | +67% | +62% | +7% | +2% | -169% | -55% |
| USA | -64% | -57% | -0% | -21% | -64% | -59% |
| AUT | +1% | -18% | +4% | -18% | -7% | +12% |
| BEL | +23% | +59% | +9% | +10% | +26% | +11% |
| BGR | -6% | -19% | -85% | -100% | -434% | -44% |
| CZE | -18% | -25% | -20% | -8% | -34% | -38% |
| DEU | +20% | +31% | -11% | -6% | -47% | -6% |
| DNK | -51% | -44% | -39% | -28% | -29% | -26% |
| ESP | +28% | +40% | -8% | -25% | -81% | -1% |
| EST | -15% | -53% | -10% | +10% | -73% | -1% |
| FIN | -84% | -196% | -12% | -16% | -27% | -38% |
| FRA | -2% | -111% | -16% | -8% | -24% | -12% |
| GBR | -64% | -4% | -13% | -49% | -45% | -24% |
| GRC | -37% | -22% | -41% | -34% | -200% | -33% |
| HRV | +26% | +39% | +5% | -16% | -108% | -8% |
| HUN | -8% | +13% | -9% | -36% | -208% | -30% |
| IRL | +4% | +37% | -9% | +35% | +42% | -8% |
| ITA | -56% | -87% | -6% | -29% | -38% | -43% |
| LTU | +51% | +52% | -44% | +8% | +63% | +18% |
| LUX | -78% | -89% | +11% | -65% | -326% | -51% |
| LVA | +18% | +25% | -29% | +18% | -173% | -56% |
| MLT | -7778% | -1410% | -603% | -410% | -597% | -512% |
| NLD | -30% | +33% | -5% | -13% | -51% | -13% |
| POL | +28% | +52% | +14% | -9% | -19% | +2% |
| PRT | -87% | -145% | -8% | -49% | -29% | -27% |
| ROU | +27% | -3% | -12% | -29% | -158% | -50% |
| RUS | +78% | +76% | -20% | +23% | -3% | -0% |
| SVK | -47% | -81% | -4% | -26% | -102% | -5% |
| SVN | -68% | -34% | -1% | -25% | -18% | +17% |
| SWE | -44% | -32% | -7% | -36% | -11% | -21% |
| CHN | -10% | -22% | +38% | -33% | -26% | +2% |
| CYP | -58% | -2% | -34% | -77% | -39% | -60% |
| IDN | +20% | -60% | -36% | -12% | -8% | -75% |
| IND | -4% | -52% | -27% | -33% | +10% | -66% |
| KOR | +10% | +78% | +76% | -8% | -29% | -8% |
| SAU | +93% | +87% | +12% | +13% | -64% | +18% |
| TUR | +9% | -14% | +4% | +24% | +2% | -20% |
| JPN | +10% | +67% | -80% | -40% | -1% | +8% |
| ZAF | +6% | -22% | +32% | +3% | +9% | -122% |

**S1.2 Air quality simulations**

The forward model simulation is the next major step in which uncertainty is introduced into our analysis. Inputs for chemical transport models, like emissions and meteorological data, have inherent biases and uncertainties; there are also uncertainties that arise due to simplifications made in the representation of physical and chemical processes. Additionally, there is uncertainty attributable to the coarseness of the spatial resolution of our simulation (2° $\times$ 2.5°, in our case). This coarse resolution will lead to underestimated exposure to anthropogenic pollution in areas that are spatially small with high levels of pollution like cities, road systems, and point sources and it will overestimate anthropogenic pollution in less polluted areas surrounding heavily polluted areas. We partially account for this coarse resolution through our use of satellite-derived concentrations in the cost-function values for PM_2.5_ which has shown good statistical agreement with observations (van Donkelaar et al., 2016); however, we do not do this for O_3_ as there is no equivalent surface-level satellite-derived product.

We compare GEOS-Chem simulated concentrations of PM_2.5_ and O_3_, along with satellite-derived PM_2.5_ concentrations (van Donkelaar et al., 2016), to the WHO’s SDG indicator (Gumy, 2022) 11.6.2 for PM_2.5_ concentrations (Figure S4a) and to ground level monitors of O_3_ from the TOAR (Schultz et al., 2017) network (Figure S4b) to characterize the uncertainty in our simulation. The SDG Indicator 11.6.2 represents concentrations of fine particulate matter (PM_2.5_) and is the annual mean values in cities in each country. For the PM_2.5_ results, we find that the satellite-derived data is better correlated with the observations and has lower bias compared to the simulated concentrations; however, both the simulated PM_2.5_ and the satellite-derived PM_2.5_ have relatively low biases and are well correlated with observations. In Figure S4b we compare simulated concentrations of the six-month peak average of maximum daily eight hour averaged O_3_ from GEOS-Chem with observations of the same O_3_ metric from the TOAR network; when considering these results, the spatial distribution of TOAR sites, which are primarily in Europe and North America, should be considered. Overall, simulated concentrations are well correlated with observations with a slight positive bias in the simulated results.


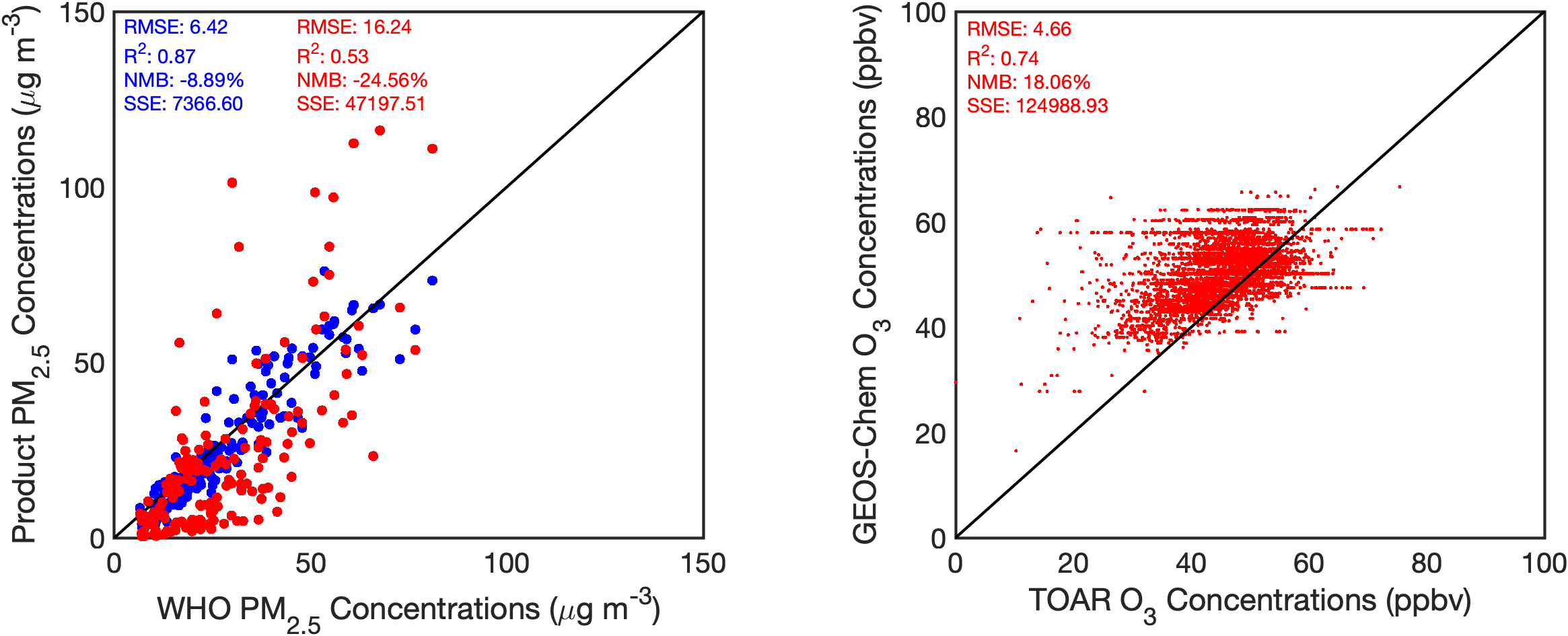


**Figure S4.** Comparison of forward model simulated PM_2.5_ concentrations to WHO indicator SDG 11.6.2 (left) and O_3_ concentrations to observations from the TOAR network (right). Red indicates GEOS-Chem simulated concentration and blue indicates satellite-derived concentrations.

**S1.3 Adjoint sensitivity analysis**

The third major step of our analysis, the use of adjoint sensitivities for source attribution and scenario estimation, introduces additional uncertainty; the next section of this supplemental consider this. The adjoint model calculates the local-linear sensitivity of a pollutant to its chemical precursor emissions, i.e., the linear response in pollutant concentrations with respect to emissions at some specific emissions magnitude. This local-linear sensitivity is unable to accurately mimic the non-linear response of both PM_2.5_ and O_3_ to large emissions perturbations. More specifically, for O_3_, one study (Cohan et al., 2005) characterized the error from first order projections associated with a -10%, -50%, and -100% change in anthropogenic NO_x_ emissions to be 4.8%, 15.9% and 29.9%, respectively. For PM_2.5­_, one study (Clappier et al., 2021) found that emission reductions of NH_3_, NO_x_, and SO_x_ of 25% led to generally linear changes in concentrations with 50% reductions incurring non-linearity, generally, less than 30%. Owing to this, when local-linear sensitivities are applied to emissions with much different magnitudes, possibly in entirely different chemical regimes, uncertainty is introduced from the adjoint’s linear projection compared to the non-linear system represented by the forward model.

We evaluate the uncertainty in this first order sensitivity calculation by performing finite difference tests. In a finite difference test, two simulations are performed: the base simulation with the same emissions used in the forward model and adjoint calculations and an additional simulation in which emissions are reduced by some percentage. By differencing the pollutant concentrations output from these two simulations in the cost-function region and applying the same operators we apply to define the adjoint cost-function, we can project the change in a cost-function from a forward model simulation. If we then perform an adjoint projection using the same change in emissions we can compare the local-linear adjoint projection to the forward model finite difference. In this case of the following analysis, we increase emissions by 20%.

In Figure S5 we present a comparison between the finite difference and adjoint projected concentrations using both HTAP and ECLIPSE emissions for PM_2.5_ for all G20 countries. Considering a 20% change in emissions, the adjoint sensitivities capture responses in the forward model well; the adjoint projection for the base HTAP simulation only has a +1% NMB. In this case, the adjoint projections are well correlated with the finite difference responses. We additionally apply adjoint projections calculated using HTAP emissions to emission perturbations using ECLIPSE to characterize the added uncertainty of performing an adjoint projection using emissions different from what were used in the forward model. As seen by comparing the left and right panels of Fig S5, we find that this only slightly degrades the performance of the adjoint projection compared to finite difference simulations. We present finite difference tests for individual grid cells for O_3_ in the main text section 3.1.


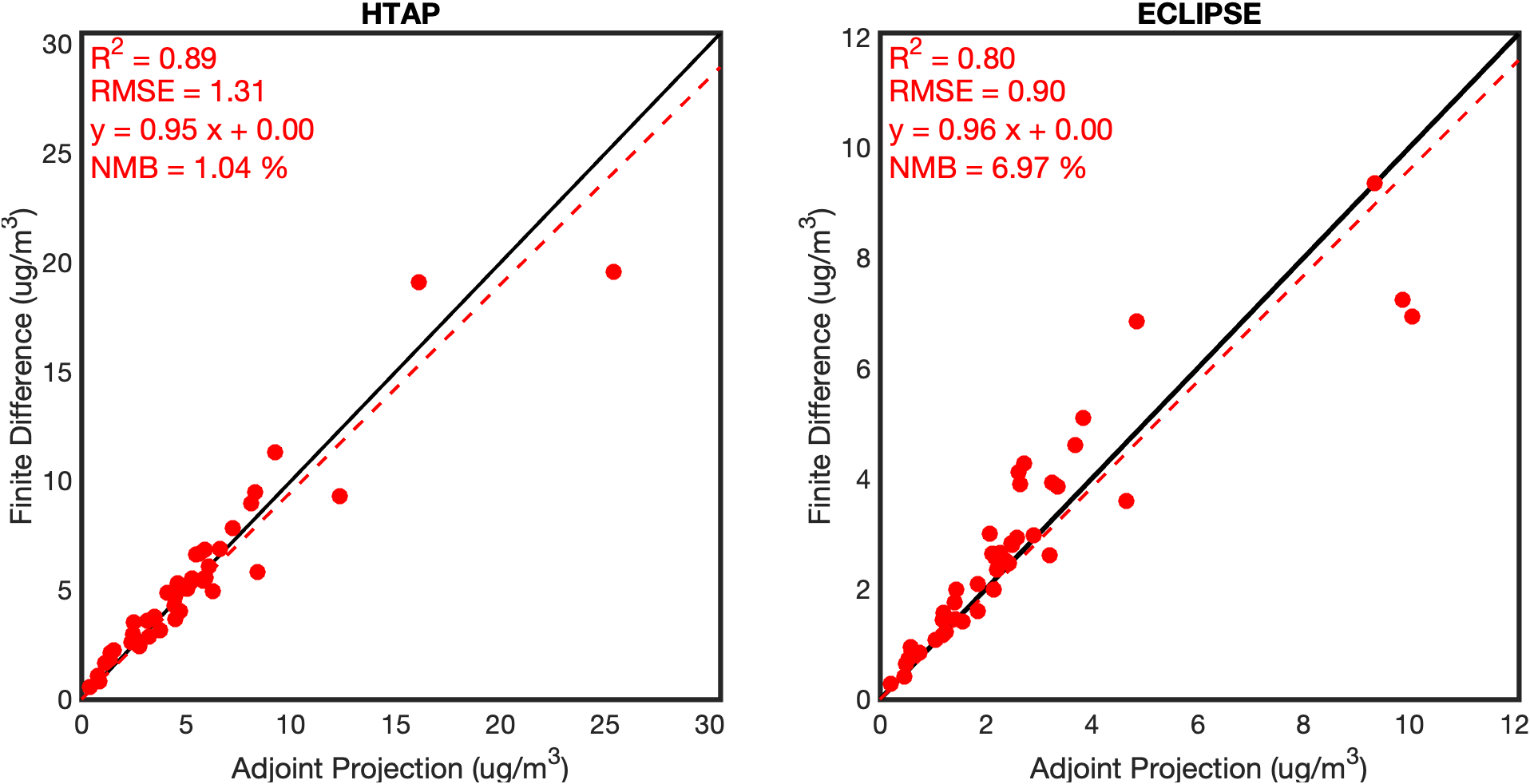


**Figure S5.** The performance of the local-linear adjoint projection for perturbations across all emission precursor species compared to a forward model finite difference test involving a 20% increase in emissions for HTAP (left) and ECLIPSE (right)

We account for the uncertainty introduced from linear adjoint projections by scaling the sum of “semi-normalized sensitivities” to the cost-function value for each adjoint simulation. The adjoint model calculates “semi-normalized sensitivities” which are equivalent to the emissions scaled sensitivities multiplied by the species total emissions used to drive the forward model. By totaling semi-normalized sensitivities across all species and sources we are able to calculate the total amount of pollution accounted for by the local linear sensitivity calculation; this is not equivalent to the cost-function, so we scale the sensitivities to sum to this value.

The adjoint sensitivities calculated for O_3_ are done so with respect to a cost-function of the six-month peak averaged daily one-hour max O_3_ concentrations (MDA1) as this metric corresponded to the relative risk exposure relationship developed in an early epidemiological study (Jerrett et al., 2009). After beginning the adjoint calculations for this work, newer health impact assessment methods for O_3_ were developed using a different O_3_ exposure metric: the six-month peak averaged daily eight-hour max O_3_ concentrations (MDA8). To align the adjoint coefficients with the most up-to-date health impact assessment method we calculate both the MDA1 and MDA8 O_3_ concentrations from the forward model and scale the MDA1 O_3_ cost-functions to the MDA8 values. In order to get an idea of the uncertainty this introduces to our final results, we tested the impact of this change in a single country, USA, and found that performing this scaling in place of defining the cost-function to be MDA8 O_3_ only led to NMB values between the MDA8 and MDA1 sensitivities of +4.8%, -4.9%, and -6.6% for NO_x_, VOCs, and CO, respectively.

One additional scaling is performed when applying the adjoint sensitivities to the emission scenario impact assessments; we scale the satellite-derived PM_2.5_ concentrations(van Donkelaar et al., 2016) to values used in the GBD study(Murray et al., 2020) by again considering results in the USA. Calculating sensitivities around satellite-derived concentrations and scaling them, compared to calculating sensitivities with updated GBD values, introduces normalized mean biases of +26.3%, +25.5%, +26.2%, +22.8%, and +27.4% for PM_2.5_ sensitivities to BC, OC, NH_3_, NO_X_, and SO_2_, respectively.

**S1.4 Health impact assessment**

We estimate uncertainty in the health impact assessment by considering the upper and lower bound values of the key components that are used in the assessment: the relative risk values, counterfactual values, population data, and baseline mortality rates. All of these lower and upper bounds are provided in the GBD study.(Murray et al., 2020)

When considering PM_2.5­_-related premature deaths in individual receptor countries, uncertainties were as high as ±56% when source contributions were aggregated across all sectoral, species-specific, and country sources, when considering uncertainty in individual source groups uncertainty in the health impact assessment is even higher. Across all G20 countries, the average uncertainty range in PM_2.5_-related premature deaths was ±23%. When considering O_3_-related premature deaths in individual receptor countries, uncertainties were as high as ±100%. Across all G20 countries the average uncertainty range in O_3_-related premature deaths was ±67%. We note that these uncertainty ranges are higher than uncertainty introduce from the linear adjoint projection assumption, the adjoint sensitivity scaling, and are also larger than the normalized mean biases calculated when comparing forward model simulated concentrations to observations. For the latter, we note that these biases include uncertainty introduced to the forward model from inputs like emissions data and metrological data. Ultimately, it is for this reason that we only report uncertainty in the additional supplementals from the health impact assessment. While we report health impact assessment uncertainty for each source receptor relationship, it should be noted that there is additional uncertainty introduced from all steps of the analysis that is not considered explicitly in the lower and upper bounds.

**S1.5 Map of G20 member states**

**
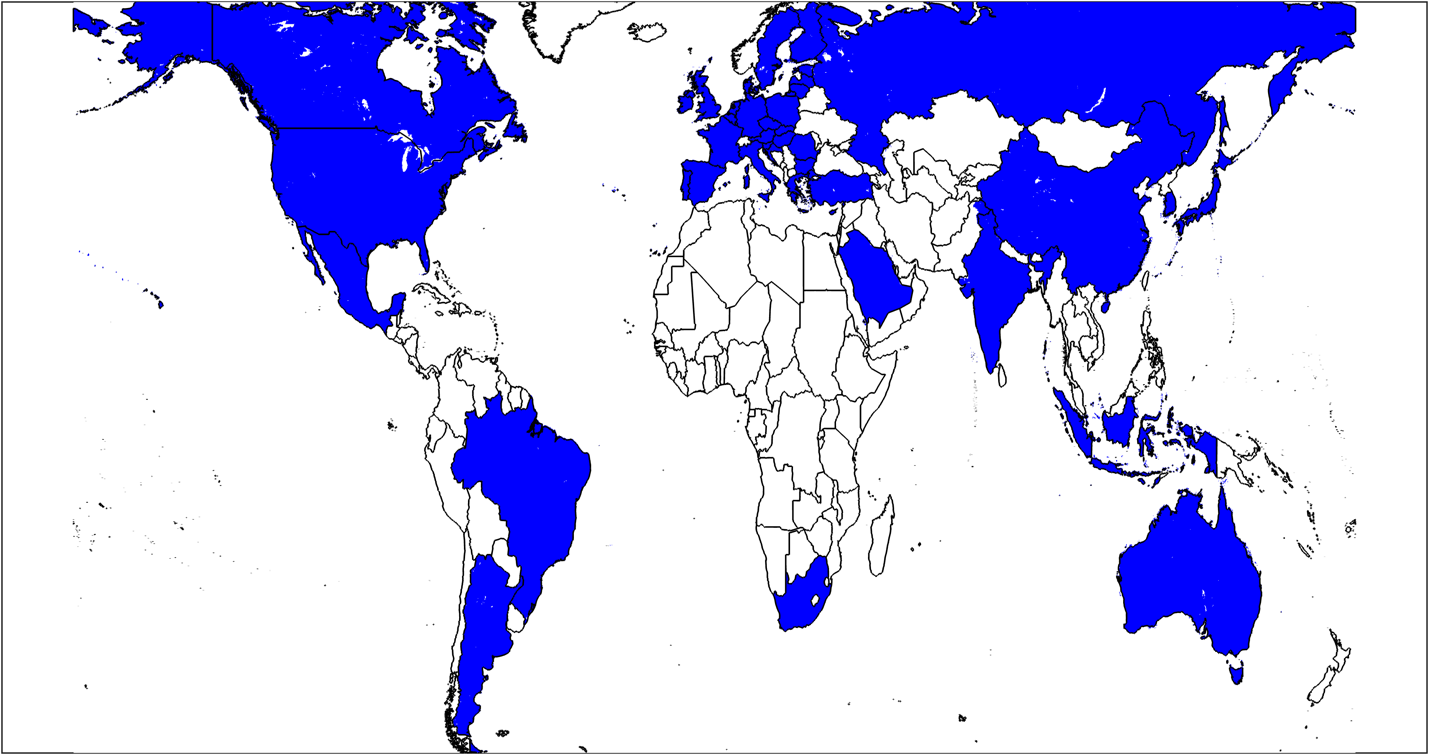
**

**Figure S6.** Map of G20 countries and member states of the European Union indicated in blue.

**S1.6 ISO-3 code mapping**

**Table S2. A mapping of ISO-3 codes to country names for all countries and regions considered in our analysis**

| **ISO-3** | **Names** |
| --- | --- |
| ARG | Argentina |
| BOL | Bolivia |
| BRA | Brazil |
| CHL | Chile |
| COL | Colombia |
| ECU | Ecuador |
| FLK | Falkland Islands |
| GUF | French Guiana |
| GUY | Guyana |
| PER | Peru |
| PRY | Paraguay |
| SUR | Suriname |
| URY | Uruguay |
| VEN | Venezuela |
| ASM | American Samoa |
| AUS | Australia |
| COK | Cook Islands |
| FJI | Fiji |
| FSM | Micronesia |
| GUM | Guam |
| KIR | Kiribati |
| MHL | Marshall Islands |
| MNP | Northern Mariana Islands |
| NCL | New Caledonia |
| NFK | Norfolk Island |
| NIU | Niue |
| NRU | Nauru |
| NZL | New Zealand |
| PCN | Pitcairn |
| PLW | Palau |
| PNG | Papua New Guinea |
| PYF | French Polynesia |
| SLB | Solomon Islands |
| TKL | Tokelau |
| TON | Tonga |
| TUV | Tuvalu |
| VUT | Vanuatu |
| WLF | Wallis and Futuna |
| WSM | Western Samoa |
| ABW | Aruba |
| AIA | Anguilla |
| ANT | Netherland Antilles |
| ATG | Antigua and Barbuda |
| BHS | Bahamas |
| BLZ | Belize |
| BMU | Bermuda |
| BRB | Barbados |
| CAN | Canada |
| CRI | Costa Rica |
| CUB | Cuba |
| CYM | Cayman Islands |
| DMA | Commonwealth of Dominica |
| DOM | Dominican Republic |
| GLP | Guadeloupe |
| GRD | Grenada |
| GRL | Greenland |
| GTM | Guatemala |
| HND | Honduras |
| HTI | Haiti |
| JAM | Jamaica |
| KNA | Saint Kitts and Nevis |
| LCA | Saint Lucia |
| MEX | Mexico |
| MSR | Montserrat |
| MTQ | Martinique |
| NIC | Nicaragua |
| PAN | Panama |
| PRI | Puerto Rico |
| SLV | El Salvador |
| SPM | Saint Pierre and Miquelon |
| TCA | Turks and Caicos Islands |
| TTO | Trinidad and Tobago |
| USA | United States of America |
| VCT | Saint Vincent |
| VGB | British Virgin Islands |
| VIR | United States Virgin Islands |
| ALB | Albania |
| AND | Andorra |
| AUT | Austria |
| BEL | Belgium |
| BGR | Bulgaria |
| BIH | Bosnia and Herzegovina |
| BLR | Belarus |
| CHE | Switzerland |
| CZE | Czech Republic |
| DEU | Germany |
| DNK | Denmark |
| ESP | Spain |
| EST | Estonia |
| FIN | Finland |
| FRA | France |
| FRO | Faeroe Islands |
| GBR | United Kingdom |
| GGY | Guernsey |
| GIB | Gibraltar |
| GRC | Greece |
| HRV | Croatia |
| HUN | Hungary |
| IRL | Ireland |
| ITA | Italy |
| JEY | Jersey |
| LIE | Liechtenstein |
| LTU | Lithuania |
| LUX | Luxembourg |
| LVA | Latvia |
| MCO | Monaco |
| MDA | Moldova |
| MKD | Macedonia |
| MLT | Malta |
| NLD | Netherlands |
| NOR | Norway |
| POL | Poland |
| PRT | Portugal |
| ROU | Romania |
| RUS | Russia |
| SCG | Serbia and Montenegro |
| SJM | Svalbard |
| SMR | San Marino |
| SVK | Slovakia |
| SVN | Slovenia |
| SWE | Sweden |
| UKR | Ukraine |
| ISL | Iceland |
| IMN | Isle of Man |
| AFG | Afghanistan |
| ARE | United Arab Emirates |
| ARM | Armenia |
| AZE | Azerbaijan |
| BGD | Bangladesh |
| BHR | Bahrain |
| BRN | Brunei Darussalam |
| BTN | Bhutan |
| CHN | China |
| CYP | Cyprus |
| GEO | Georgia |
| HKG | Hong Kong |
| IDN | Indonesia |
| IND | India |
| IRN | Iran |
| IRQ | Iraq |
| ISR | Israel |
| JOR | Jordan |
| KAZ | Kazakhstan |
| KGZ | Kyrgyz Republic |
| KHM | Cambodia |
| KOR | South Korea |
| KWT | Kuwait |
| LAO | Laos |
| LBN | Lebanon |
| MAC | Macao |
| MDV | Maldives |
| MMR | Myanmar |
| MNG | Mongolia |
| MYS | Malaysia |
| NPL | Nepal |
| OMN | Oman |
| PAK | Pakistan |
| PHL | Philippines |
| PRK | North Korea |
| PSE | Occupied Palestinian Territory |
| QAT | Qatar |
| SAU | Saudi Arabia |
| SGP | Singapore |
| SYR | Syria |
| THA | Thailand |
| TJK | Tajikistan |
| TKM | Turkmenistan |
| TLS | East Timor |
| TUR | Turkey |
| UZB | Uzbekistan |
| VNM | Vietnam |
| YEM | Yemen |
| JPN | Japan |
| LKA | Sri Lanka |
| TWN | Taiwan |
| AGO | Angola |
| BDI | Burundi |
| BEN | Benin |
| BFA | Burkina Faso |
| BWA | Botswana |
| CAF | Central African Republic |
| CIV | Ivory Coast |
| CMR | Cameroon |
| COD | Congo, Democratic Republic |
| COG | Congo |
| DJI | Djibouti |
| DZA | Algeria |
| EGY | Egypt |
| ERI | Eritrea |
| ETH | Ethiopia |
| GAB | Gabon |
| GHA | Ghana |
| GIN | Guinea |
| GMB | Gambia |
| GNB | Guinea-Bissau |
| GNQ | Equatorial Guinea |
| KEN | Kenya |
| LBR | Liberia |
| LBY | Libyan Arab Jamahiriya |
| LSO | Lesotho |
| MAR | Morocco |
| MLI | Mali |
| MOZ | Mozambique |
| MRT | Mauritania |
| MWI | Malawi |
| NAM | Namibia |
| NER | Niger |
| NGA | Nigeria |
| REU | Reunion |
| RWA | Rwanda |
| SDN | Sudan |
| SEN | Senegal |
| SLE | Sierra Leone |
| SOM | Somalia |
| SWZ | Swaziland |
| TCD | Chad |
| TGO | Togo |
| TUN | Tunisia |
| TZA | United Rep. of Tanzania |
| UGA | Uganda |
| ZAF | South Africa |
| ZMB | Zambia |
| ZWE | Zimbabwe |
| CPV | Cape Verde |
| STP | Sao Tome and Principe |
| SHN | Saint Helena |
| MUS | Mauritius |
| MDG | Madagascar |
| COM | Comoros |
| MYT | Mayotte |
| SYC | Seychelles |
| SRB | Serbia |
| MNE | Montenegro |
| XKX | Kosovo |

**S1.7 References**

Clappier, A., Thunis, P., Beekmann, M., Putaud, J. P., & de Meij, A. (2021). Impact of SOx, NOx and NH3 emission reductions on PM2.5 concentrations across Europe: Hints for future measure development. *Environment International*, *156*, 106699. https://doi.org/10.1016/j.envint.2021.106699

Cohan, D. S., Hakami, A., Hu, Y., & Russell, A. G. (2005). Nonlinear Response of Ozone to Emissions: Source Apportionment and Sensitivity Analysis. *Environmental Science & Technology*, *39*(17), 6739–6748. https://doi.org/10.1021/es048664m

Gumy, S. (2022). *SDG Indicator 11.6.2*. SDG Indicator 11.6.2 Concentrations of Fine Particulate Matter (PM2.5). https://www.who.int/data/gho/data/indicators/indicator-details/GHO/concentrations-of-fine-particulate-matter-(pm2-5)

IIASA. (2021, July 14). *ECLIPSE V5a global emission fields*. International Institute for Applied System Analysis. https://iiasa.ac.at/web/home/research/researchPrograms/air/ECLIPSEv5a.html

Janssens-Maenhout, G., Crippa, M., Guizzardi, D., Dentener, F., Muntean, M., Pouliot, G., Keating, T., Zhang, Q., Kurokawa, J., Wankmüller, R., Denier van der Gon, H., Kuenen, J. J. P., Klimont, Z., Frost, G., Darras, S., Koffi, B., & Li, M. (2015). HTAP_v2.2: A mosaic of regional and global emission grid maps for 2008 and 2010 to study hemispheric transport of air pollution. *Atmospheric Chemistry and Physics*, *15*(19), 11411–11432. https://doi.org/10.5194/acp-15-11411-2015

Jerrett, M., Burnett, R. T., Pope, C. A., Ito, K., Thurston, G., Krewski, D., Shi, Y., Calle, E., & Thun, M. (2009). Long-Term Ozone Exposure and Mortality. *New England Journal of Medicine*, *360*(11), 1085–1095. https://doi.org/10.1056/NEJMoa0803894

Mallet, V., & Sportisse, B. (2006). Uncertainty in a chemistry-transport model due to physical parameterizations and numerical approximations: An ensemble approach applied to ozone modeling. *Journal of Geophysical Research: Atmospheres*, *111*(D1). https://doi.org/10.1029/2005JD006149

Molod, A., Takacs, L., Suarez, M., Bacmeister, J., Song, I.-S., & Eichmann, A. (2012). *The GEOS-5 Atmospheric General Circulation Model: Mean Climate and Development from MERRA to Fortuna* (GSFC.TM.01153.2012). https://ntrs.nasa.gov/citations/20120011790

Murray, C. J. L., Aravkin, A. Y., Zheng, P., Abbafati, C., Abbas, K. M., Abbasi-Kangevari, M., Abd-Allah, F., Abdelalim, A., Abdollahi, M., Abdollahpour, I., Abegaz, K. H., Abolhassani, H., Aboyans, V., Abreu, L. G., Abrigo, M. R. M., Abualhasan, A., Abu-Raddad, L. J., Abushouk, A. I., Adabi, M., … Lim, S. S. (2020). Global burden of 87 risk factors in 204 countries and territories, 1990–2019: A systematic analysis for the Global Burden of Disease Study 2019. *The Lancet*, *396*(10258), 1223–1249. https://doi.org/10.1016/S0140-6736(20)30752-2

Nawaz, M. O., & Henze, D. K. (2020). Premature Deaths in Brazil Associated With Long-Term Exposure to PM2.5 From Amazon Fires Between 2016 and 2019. *GeoHealth*, *4*(8), e2020GH000268. https://doi.org/10.1029/2020GH000268

Nawaz, M. O., Henze, D. K., Harkins, C., Cao, H., Nault, B., Jo, D., Jimenez, J., Anenberg, S. C., Goldberg, D. L., & Qu, Z. (2021). Impacts of sectoral, regional, species, and day-specific emissions on air pollution and public health in Washington, DC. *Elementa: Science of the Anthropocene*, *9*(1), 00043. https://doi.org/10.1525/elementa.2021.00043

Schultz, M. G., Schröder, S., Lyapina, O., Cooper, O. R., Galbally, I., Petropavlovskikh, I., von Schneidemesser, E., Tanimoto, H., Elshorbany, Y., Naja, M., Seguel, R. J., Dauert, U., Eckhardt, P., Feigenspan, S., Fiebig, M., Hjellbrekke, A.-G., Hong, Y.-D., Kjeld, P. C., Koide, H., … Zhiqiang, M. (2017). Tropospheric Ozone Assessment Report, links to Global surface ozone datasets [Data set]. In *Supplement to: Schultz, MG et al. (2017): Tropospheric Ozone Assessment Report: Database and Metrics Data of Global Surface Ozone Observations. Elementa—Science of the Anthropocene, 5:58, 26 pp, https://doi.org/10.1525/elementa.244*. PANGAEA. https://doi.org/10.1594/PANGAEA.876108

van Donkelaar, A., Martin, R. V., Brauer, M., Hsu, N. C., Kahn, R. A., Levy, R. C., Lyapustin, A., Sayer, A. M., & Winker, D. M. (2016). Global Estimates of Fine Particulate Matter using a Combined Geophysical-Statistical Method with Information from Satellites, Models, and Monitors. *Environmental Science & Technology*, *50*(7), 3762–3772. https://doi.org/10.1021/acs.est.5b05833
